# Supplementary material for: Reassessing Choice Probability: What 59 Macaque Studies Tell Us About Decision-Related Activity in Visual Cortex
Source: bioRxiv. 2026 Jun 11:2026.06.08.730961. Preprint. [Version 1] doi: 10.64898/2026.06.08.730961 (PMC13278128; doi:10.64898/2026.06.08.730961)
Supplement: Supplementary file 1 [file NIHPP2026.06.08.730961v1-supplement-1.pdf]

bioRxiv preprint doi: https://doi.org/10.64898/2026.06.08.730961; this version posted June 11, 2026. The copyright holder for this preprint (which was not certified by peer review) is the author/funder, who has granted bioRxiv a license to display the preprint in perpetuity. It is made available under a CC-BY 4.0 International license.

8 Supplement

8.1 Tables

Table 1: Summary of papers used in the meta-analysis (see also full dataset).

Pts: number of data points; **Pts comb.**: number of data points for which the mean CP was estimated from pooled data across monkeys; **Monk.**: number of monkeys; **Cond.**: number of task conditions; **N/P**: whether N/P ratio was reported (if mixed, shown as "+; -"); **Stim. Dur. (s)**: stimulus duration; **Task**: task type.

Paper	Pts	Pts comb.	Monk.	Cond.	N/P	Brain Area	Stim. Dur. (s)	Task
Britten et al. (1996)	5	1	4	2	+; -	MT	2	coarse
Dodd et al. (2001)	2	0	2	1	-	MT	2	bistable
Cook and Maunsell (2002)	1	1	2	1	+	MT	0.75	detection
Grunewald et al. (2002)	2	2	2	1	-	V1; MT	1	bistable
Williams et al. (2003)	2	2	2	1	-	MT; MST/MSTd	0.8	bistable
Heuer and Britten (2004)	2	0	2	1	+	MST/MSTd	1	coarse
Krekelberg and Albright (2004)	2	0	1	2	+	MT	1	coarse
Krug et al. (2004)	1	1	2	1	-	MT	2	bistable
Uka and DeAngelis (2004)	2	0	2	1	+	MT	1.5	coarse
Liu and Newsome (2005)	1	1	2	1	+	MT	1	fine
Purushothaman and Bradley (2005)	1	1	2	1	+	MT	1	fine
Uka et al. (2005)	2	0	2	1	-	IT	1	fine
Nienborg and Cumming (2006)	3	1	2	1	+; -	V1; V2	2	coarse
Uka and DeAngelis (2006); Gazzaniga (2009) Chapter 33	2	0	2	1	+	MT	1.5	fine
Gu et al. (2007)	2	0	2	1	+	MST/MSTd	2	fine
Maier et al. (2007)	2	1	2	2	-	MT	0.8	bistable
Mruczek and Sheinberg (2007)	2	2	2	2	-	IT		coarse
Nienborg and Cumming(2007)	1	0	1	1	-	V2	2	coarse
Palmer et al. (2007)	1	1	3	1	+	V1	0.2	detection
Gu et al. (2008)	4	0	2	2	+	MST/MSTd	2	fine
Law and Gold (2008)	1	1	2	1	+	MT	0.74	coarse

bioRxiv preprint doi: https://doi.org/10.64898/2026.06.08.730961; this version posted June 11, 2026. The copyright holder for this preprint (which was not certified by peer review) is the author/funder, who has granted bioRxiv a license to display the preprint in perpetuity. It is made available under a CC-BY 4.0 International license.

Paper	Pts	Pts comb.	Monk.	Cond.	N/P	Brain Area	Stim. Dur. (s)	Task
Matsumora et al. (2008)	2	0	2	1	+	IT	0.5	fine
Thiele and Hoffmann (2008)	1	1	2	1	-	MT		coarse
Cohen and Newsome (2009)	3	1	2	2	+; -	MT	0.585; 0.63; 0.674	coarse
Ghose and Harrison 2009	1	1	2	1	-	MT	0.0715	detection
Herrington et al. (2009)	1	1	2	1	-	MT	0.15	detection
Nienborg and Cumming (2009)	1	1	2	1	-	V2	2	coarse
Sasaki and Uka (2009)	2	2	2	2	+	MT	0.5	coarse
Price and Born (2010)	2	0	2	1	-	MT		fine
Verhoef et al. (2010)	2	0	2	1	-	IT	0.8	coarse
Adab and Vogels (2011)	2	0	2	1	+	V4	0.25	coarse
Bosking and Maunsell (2011)	2	2	2	2	-	MT	0.5	detection
Smith et al. (2011)	1	1	2	1	-	MT	0.2	detection
Rao et al. (2012)	1	0	1	1	-	MT	1	coarse
Shiozaki et al. (2012)	2	0	2	1	+	V4	1.5	fine
Uka et al. (2012)	4	0	4	2	+	MT	1.5	coarse
Kumano and Uka (2014)	2	0	2	1	+	MT	0.5	coarse
Nienborg and Cumming (2014)	2	0	2	1	+	V1	2	coarse
Xu et al. (2014)	2	0	2	1	+	MST/MSTd	0.6	fine
Kim et al. (2015)	2	0	2	1	+	MT	2	coarse
Smolyanskaya et al. (2015)	4	0	2	2	-	MT		detection
Krug et al. (2016)	4	0	2	2	+	MT	2	bistable
Clery et al. (2017)	2	0	2	1	+	V2	2	fine
Goris et al. (2017)	4	0	2	1	+	V1; V2	0.5	fine
Yates et al. (2017)	1	1	2	1	-	MT	1.05	coarse
Zaidel et al. (2017)	4	0	2	2	+	MST/MSTd	1	fine
Bondy et al. (2018)	2	0	2	1	-	V1	2	coarse
Sanayei et al. (2018)	2	0	2	1	-	V4	0.512	fine
Yu and Gu (2018)	7	0	2	2	+	MT; MST/MSTd	1	coarse; fine
Elmore et al. (2019)	4	0	3	1	+	V3A; CIP	1	fine
Kang and Maunsell (2020)	5	3	2	2	+; -	V1; MT	0.15	detection

bioRxiv preprint doi: https://doi.org/10.64898/2026.06.08.730961; this version posted June 11, 2026. The copyright holder for this preprint (which was not certified by peer review) is the author/funder, who has granted bioRxiv a license to display the preprint in perpetuity. It is made available under a CC-BY 4.0 International license.

Paper	Pts	Pts comb.	Monk.	Cond.	N/P	Brain Area	Stim. Dur. (s)	Task
Chang et al. (2020); Doudlah et al. (2022)	5	0	3	1	-	V3A; CIP	1	coarse
Quinn et al. (2021)	6	2	2	2	-	V2; V3/V3A	2	coarse
Clark and Bradley (2022)	2	0	2	1	+	MT	1.5	bistable
Kim et al. (2022)	2	0	2	1	-	MT	2.1	detection
Lange et al. (2023)	4	0	2	2	+	V1	1.224	coarse
Levi et al. (2023)	6	0	2	3	-	MT	1.05	coarse
Laamerad et al. (2024)	6	0	2	3	+	MT	0.067	coarse
Ziemba et al. (2024)	4	0	2	1	+	V1; V2	0.5	fine

Table 2: Explanation of terms used in Fig. 2 and Fig. S1

Terms	Description
Decoder type	Characteristics of the assumed readout mechanism used to estimate the stimulus category from neural responses – including its linearity, optimality, and the composition of the readout pool (Shadlen et al., 1996; Haefner et al., 2013; Pitkow et al., 2015)
Read out	Weight profile of the decoder – the contribution of each neuron in the readout pool to the decoder's output
Covariability	The trial-to-trial covariability in the responses of sensory neurons when the stimulus is fixed; commonly referred to as noise correlations (Kohn et al., 2016)
Sensitivity	A measure of how accurately a neuron discriminates between two stimulus categories in a task (Britten et al., 1992)
Brain area	Functional and anatomical brain region from which neural activity was recorded and CP was calculated (Kravitz et al., 2011)
Decision confidence	Subjective certainty about the choice (Pouget et al., 2016)
Engagement [3]	Level of subject's involvement and motivation during the task Aston-Jones and Cohen (2005)
Task learning [3]	How well the subject has learned the task-relevant stimulus–choice mapping (Gold and Shadlen, 2007)
Perceptual learning [3]	Improvement in the ability to discriminate or detect sensory stimuli through practice or experience (Gilbert et al., 2001; Sagi, 2011)

Continued on next page

[3] Grouped under the "Learning and Engagement" category in Fig. 2.

bioRxiv preprint doi: https://doi.org/10.64898/2026.06.08.730961; this version posted June 11, 2026. The copyright holder for this preprint (which was not certified by peer review) is the author/funder, who has granted bioRxiv a license to display the preprint in perpetuity. It is made available under a CC-BY 4.0 International license.

**Terms**	**Description**
Tailoring stimulus	The degree to which different stimulus properties were adjusted to match the tuning characteristics of recorded neurons (Britten et al., 1992)
Task type	Classification of perceptual decision tasks: 1. Coarse-discrimination 2. Fine-discrimination 3. Detection 4. Stimulus with bistable perception
Stimulus duration	Duration for which the stimulus is presented during a trial
Lapse rate	Proportion of errors on very perceptually easy trials (Prins, 2012; McCarley and Yamani, 2021)
Task exposure	Amount of time the subject spent on the task before and during data collection
Neuronal preferences	How neuron's preferences used in CP calculation were derived: 1. From task trials 2. From separate passive viewing sessions
Psych. (psychometric) kernel [4]	Weights that quantify the contribution of different stimulus features to the subject's choice (Nienborg and Cumming, 2009)
Reward scheme	The specific structure of reinforcement provided during the task, including the magnitude, probability, and symmetry of payoffs across different choices (Rorie et al., 2010)
Confounds	Factors not directly related to stimulus processing or decision-making that can nonetheless alter CP estimates
Saccade planning	Motor preparation signals related to eye movements to a choice target (Sommer and Wurtz, 2008)
Frozen vs random seed	CP is calculated using only trials with truly identical stimuli vs. stimuli with varying noise (Britten et al., 1996)
Zero signal vs grand CP	CP is calculated using only trials with truly ambiguous stimuli vs. combining z-scored responses from several signal strengths (Britten et al., 1996)
Control of eye movements	Methods that control for small movements of the eyes during fixation that cause variability in the retinal image (Gur et al., 1997; Bair and O'Keefe, 1998)
Stimulus and targets location	Position of the stimulus and choice targets relative to fixation point
Predictability of saccade targets	Quantified as the consistency or variability in target locations across trial and days (Basso and Wurtz, 1997)

1824

[4] This and all subsequent categories are presented exclusively in Fig. S1.

bioRxiv preprint doi: https://doi.org/10.64898/2026.06.08.730961; this version posted June 11, 2026. The copyright holder for this preprint (which was not certified by peer review) is the author/funder, who has granted bioRxiv a license to display the preprint in perpetuity. It is made available under a CC-BY 4.0 International license.

Table 3: Annotation of the arrows in Fig. 2 and Fig. S1

The "Sign" column indicates the expected direction of influence. A missing sign means that the direction cannot be specified, either because the relationship is non-monotonic or because one of the variables lacks a hierarchical ordering.

**Parent → Child**	**Sign**	**Rationale based on the literature**
Decoder type/Read out → Choice correlations		In the feedforward paradigm, the weight profile of the decoder influences CP (see Equation (1)).
Covariability → Choice correlations	+	Holds both for the feedforward (Haefner et al., 2013) and feedback (Haefner et al., 2016; Chicharro et al., 2021) paradigms
Sensitivity → Read out	+	In near-optimal decoders or inference models, neurons with higher task sensitivity tend to have stronger impact on the decision, though this link is modulated by the correlation structure among neurons in the decision pool (Haefner et al., 2013)
Brain area → Decoder type-/Read out		The brain area's position in the hierarchy and its relevance to the task determine how and whether the decoder utilizes this area for the decision (Kang and Maunsell, 2020)
Brain area → Covariability		Covariability may differ between brain areas. The strongest effects are expected in brain regions that directly receive feedback from the decision area, with weaker influence in more distant regions
Brain area → Sensitivity		The neural sensitivity for a task depends on the brain area
Decision confidence → Covariability	+	In hierarchical inference models the strength of the top-down belief signal that shapes covariability increases with confidence (Haefner et al., 2016; Lange and Haefner, 2022)
Engagement [3] → Covariability	+	Task engagement may influence the strength of covariability (Ruff and Cohen, 2014; Ni et al., 2018)
Engagement [3] → Sensitivity	+	Increased engagement may enhance neuronal sensitivity via feedback mechanisms associated with feature-based attention and alertness (Reynolds and Chelazzi, 2004; Mitchell et al., 2007)
Engagement [3] → Lapse rate	−	Fewer inattentive errors due to heightened attention (McGinley et al., 2015)
Task learning [3] → Decoder type/Read out	+	Makes decoder/read-out closer to optimal (Law and Gold, 2008)
Task learning → Covariability	+	Has been hypothesized/found to influence covariability (Haefner et al., 2016; Ni et al., 2018; Lange and Haefner, 2022)

Continued on next page

bioRxiv preprint doi: https://doi.org/10.64898/2026.06.08.730961; this version posted June 11, 2026. The copyright holder for this preprint (which was not certified by peer review) is the author/funder, who has granted bioRxiv a license to display the preprint in perpetuity. It is made available under a CC-BY 4.0 International license.

**Parent → Child**	**Sign**	**Rationale based on the literature**
Task learning [3] → Lapse rate	−	Better understanding of task structure reduces errors on easy trials
Perceptual learning [3] → Covariability	−	Has been hypothesized/found to influence covariability (Ni et al., 2018)
Perceptual learning [3] → Sensitivity	+	Has been found to increase task sensitivity of the neurons, thus improving stimulus encoding (Schoups et al., 2001; Yan et al., 2014; Sagi, 2011)
Stimulus duration → Covariability	+	Longer stimulus durations may allow more time for feedback or inference-related signals to modulate covariability (Haefner et al., 2016; Nienborg and Cumming, 2009)
Tailoring stimulus → Sensitivity	+	Stimuli matched to a neuron's tuning has been found to increase its task sensitivity (Britten et al., 1992, 1996)
Task type → Tailoring stimulus		The complexity and demands of each task type may affect the researcher's strategy for tailoring stimulus parameters
Task type → Sensitivity		Lower sensitivity tasks have been found in fine- than in coarse-discrimination tasks(Purushothaman and Bradley, 2005; Uka and DeAngelis, 2006; Xu et al., 2014; Kim et al., 2015)
Task type → Decoder type-/Read out		In detection tasks, the brain may rely on a one-pool decoder, whereas two-pool models are more consistent with discrimination tasks (Nienborg et al., 2012).
Task type → Decision confidence		Stimuli with bistable perception may produce higher decision confidence (Bainbridge et al., 2015)
Task exposure → Task learning [3]	+	Longer exposure may lead to refinement of the subject's strategy toward optimal
Task exposure → Perceptual learning [3]	+	Enables the cortical plasticity that drives perceptual learning by repeatedly activating task-relevant neural circuits over time (Schoups et al., 2001)
Engagement [3] → Psych. kernel [4]	+	Increases the amplitude of the psychophysical kernel as the subject becomes more sensitive to task-relevant stimulus features
Task learning [3] → Psych. kernel	+	Make the shape of the kernel closer to the task structure (Nienborg and Cumming, 2009; Lange et al., 2023; Liu et al., 2026)
Reward scheme → *Engagement* [3]	+	Higher anticipated reward magnitudes increase task motivation and global arousal (Maunsell, 2004)
Confounds → Choice correlation		Overestimate or underestimate the measure if not appropriately addressed

Continued on next page

bioRxiv preprint doi: https://doi.org/10.64898/2026.06.08.730961; this version posted June 11, 2026. The copyright holder for this preprint (which was not certified by peer review) is the author/funder, who has granted bioRxiv a license to display the preprint in perpetuity. It is made available under a CC-BY 4.0 International license.

**Parent → Child**	**Sign**	**Rationale based on the literature**
Stimulus duration → Confounds	+	Longer stimulus has hypothesized to improve the signal-to-noise ratio in Poisson-like neurons, enhancing choice discriminability and reducing CP underestimation (Kang and Maunsell, 2012)
Frozen vs random seed → Confounds	+	The additional variability of the stimulus created by random noise have been found to inflate the estimates (Nienborg and Cumming, 2009)
Zero signal vs grand CP → Confounds	+/-	Grand CP can either inflate CP by incompletely removing stimulus effects, or underestimate it by eliminating real choice signals through z-scoring of unbalanced data (Kang and Maunsell, 2012)
Control of eye movements → Confounds		Variability in eye position alters sensory input; if correlated with choice, this can bias CP estimates (Herrington et al., 2009). Because studies control for these movements to varying degrees, this may create cross-study variability in mean CP
Saccade planning → Choice Correlation		Planned saccades has been found to enhance the responses of neurons when the choice target is near their receptive field, potentially increasing choice correlations in tasks with asymmetric target positions (Laamerad et al., 2024)
Stimulus and targets location → Saccade planning		Determine the planned saccade endpoint relative to each neuron's receptive field
Predictability of saccade targets → Saccade planning	+	Predictable target locations facilitate early saccade planning (Zhang and Gu, 2026)
Choice Correlation → Mean CP		Choice correlations reflect the magnitude of CP relative to 0.5 (Haefner et al., 2013; Pitkow et al., 2015)
Neuronal preferences → Mean CP		The sign of CP – whether it higher or lower than 0.5 – is defined with respect to a neuron's preferred stimulus. Estimating that preference from task trials may inflate CP for neurons with low feedforward sensitivity (Zaidel et al., 2017)

bioRxiv preprint doi: https://doi.org/10.64898/2026.06.08.730961; this version posted June 11, 2026. The copyright holder for this preprint (which was not certified by peer review) is the author/funder, who has granted bioRxiv a license to display the preprint in perpetuity. It is made available under a CC-BY 4.0 International license.

Table 4: Linear regression results predicting mean CP using inverse mean N/P ratio, brain area, stimulus duration, and task type.

**Predictor**	**Estimate**	**Std. Error**	**t value**	**Pr($>|t|$)**
(Intercept)	0.4789	0.0145	33.04	$< 2 \times 10^{-16}$ ***
inverse NP.ratio	0.0435	0.0188	2.31	0.0237 *
Brain.areasV2	0.0168	0.0175	0.96	0.3390
Brain.areasV4	0.0259	0.0200	1.30	0.1993
Brain.areasIT	0.0188	0.0282	0.67	0.5071
Brain.areasV3/V3A	-0.0126	0.0271	-0.47	0.6431
Brain.areasMT	0.0261	0.0116	2.26	0.0266 *
Brain.areasMST/MSTd	0.0157	0.0135	1.16	0.2501
Brain.areasCIP	0.0723	0.0271	2.67	0.0094 **
Stimulus duration (s)	0.0223	0.0065	3.43	0.0010 **
Task: fine-discrimination	-0.0058	0.0106	-0.54	0.5903
Task: detection	0.0307	0.0180	1.70	0.0933 .
Task: bistable	0.0357	0.0167	2.14	0.0360 *

Residual Std. Error: 0.0344 on 75 df
Multiple R-squared: 0.4178, *Adjusted R-squared:* 0.3247
F-statistic: 4.486 on 12 and 75 df, *p-value:* 2.16×10^{-5}

bioRxiv preprint doi: https://doi.org/10.64898/2026.06.08.730961; this version posted June 11, 2026. The copyright holder for this preprint (which was not certified by peer review) is the author/funder, who has granted bioRxiv a license to display the preprint in perpetuity. It is made available under a CC-BY 4.0 International license.

Table 5: Linear regression results predicting mean CP using tailoring-based sensitivity proxy, brain area, stimulus duration, and task type.

**Predictor**	**Estimate**	**Std. Error**	**t value**	**Pr(> $\|t\|$)**
(Intercept)	0.4912	0.0192	25.59	$< 2 \times 10^{-16}$ ***
Task_parm: population	-0.0324	0.0153	-2.12	0.0360 *
Task_parm: single neuron	0.0056	0.0105	0.53	0.5947
Non_task_parm: population	0.0036	0.0135	0.27	0.7871
Non_task_parm: single neuron	0.0105	0.0107	0.98	0.3284
Stimulus size: fit to population	0.0058	0.0194	0.30	0.7659
Stimulus size: fit to RF	-0.0071	0.0188	-0.38	0.7042
Stimulus size: smaller than RF	0.0028	0.0216	0.13	0.8988
Brain.areasV2	0.0388	0.0157	2.47	0.0149 *
Brain.areasV4	0.0468	0.0203	2.31	0.0226 *
Brain.areasIT	0.0374	0.0247	1.51	0.1332
Brain.areasV3/V3A	0.0292	0.0199	1.47	0.1438
Brain.areasMT	0.0369	0.0129	2.87	0.0049 **
Brain.areasMST/MSTd	0.0244	0.0183	1.34	0.1838
Brain.areasCIP	0.0869	0.0255	3.41	0.0009 ***
Stimulus duration (s)	0.0194	0.0060	3.22	0.0016 **
Task: fine-discrimination	-0.0212	0.0092	-2.31	0.0226 *
Task: detection	0.0235	0.0129	1.82	0.0711 .
Task: bistable	0.0434	0.0133	3.26	0.0015 **

Residual Std. Error: 0.0374 on 121 df
Multiple R-squared: 0.4276, *Adjusted R-squared:* 0.3424
F-statistic: 5.021 on 18 and 121 df, *p-value:* 2.33×10^{-8}

bioRxiv preprint doi: https://doi.org/10.64898/2026.06.08.730961; this version posted June 11, 2026. The copyright holder for this preprint (which was not certified by peer review) is the author/funder, who has granted bioRxiv a license to display the preprint in perpetuity. It is made available under a CC-BY 4.0 International license.

1825 ## 8.2 Figures

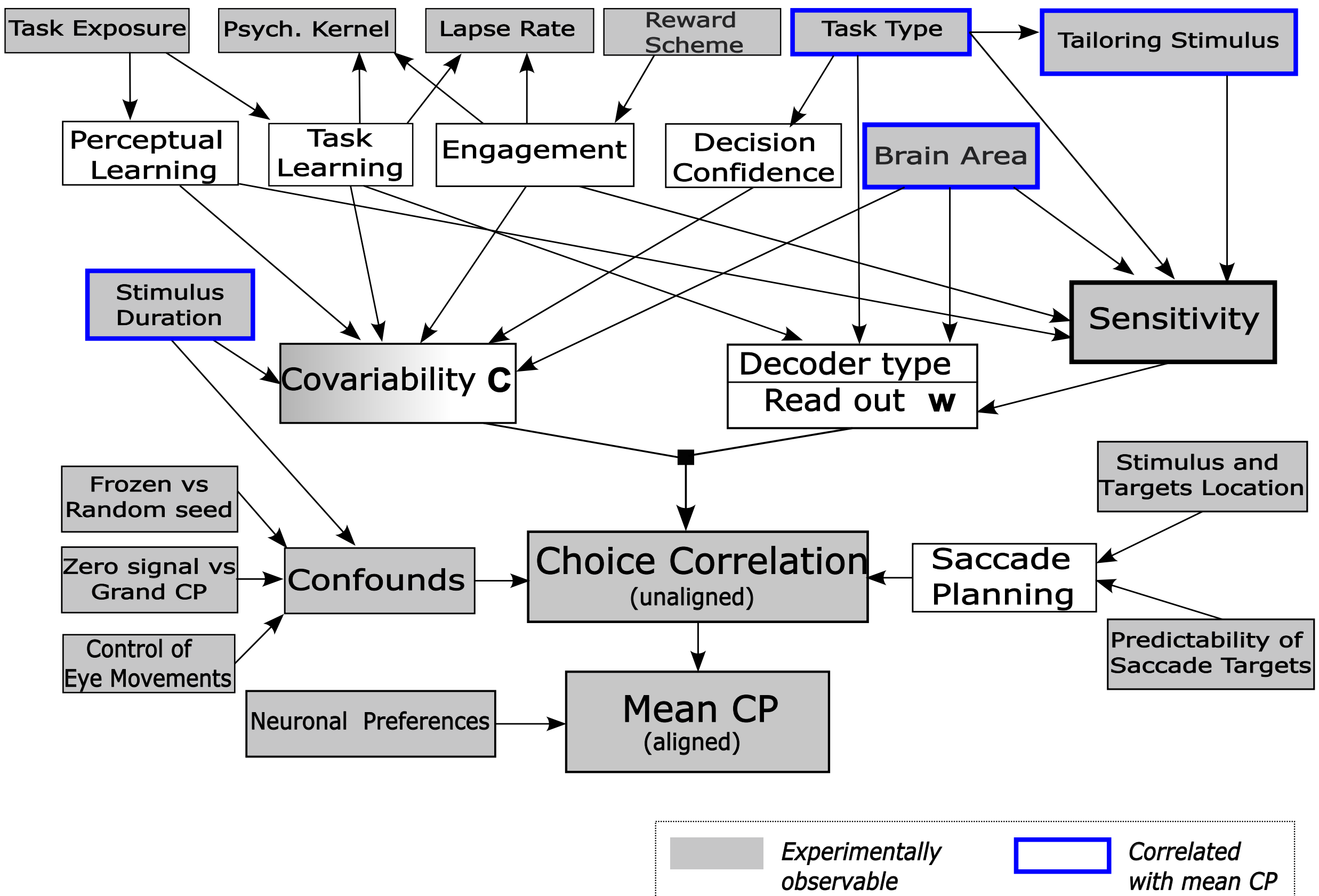

Figure S1: **Expanded schematic of hypothesized factors influencing Choice Probability (CP).** This supplementary diagram extends the framework presented in Fig. 2 by incorporating additional elements: (1) potential confounds and pre-motor signals, and (2) experimental factors that could not be evaluated in this meta-analysis due to data limitations (Psych. Kernel, Reward Scheme). It was proposed that at least part of the observed choice correlations may be explained by pre-motor signals during saccade planning (Laamerad et al., 2024; Zhang and Gu, 2026)(lower right part of the diagram). Methodological choices that date back to Britten et al. (1996) such as CP estimation procedures ("Grand CP") and stimulus control techniques ("frozen noise", eye movement control) may influence CP estimates (Nienborg and Cumming, 2009; Kang and Maunsell, 2012; Herrington et al., 2009) (lower left of the diagram). Additionally, compared to Fig. 2 the "Learning and Engagement" node has been subdivided into "Task learning," "Perceptual learning," and "Engagement." Arrows denote putative causal relationships between experimental design choices, intermediate variables, and core factors driving CP (see Tables 2 and 3 for details). Gray boxes denote experimentally observable variables. Thick blue borders highlight variables that exhibited a statistically significant relationship with mean CP in our analysis.

bioRxiv preprint doi: https://doi.org/10.64898/2026.06.08.730961; this version posted June 11, 2026. The copyright holder for this preprint (which was not certified by peer review) is the author/funder, who has granted bioRxiv a license to display the preprint in perpetuity. It is made available under a CC-BY 4.0 International license.

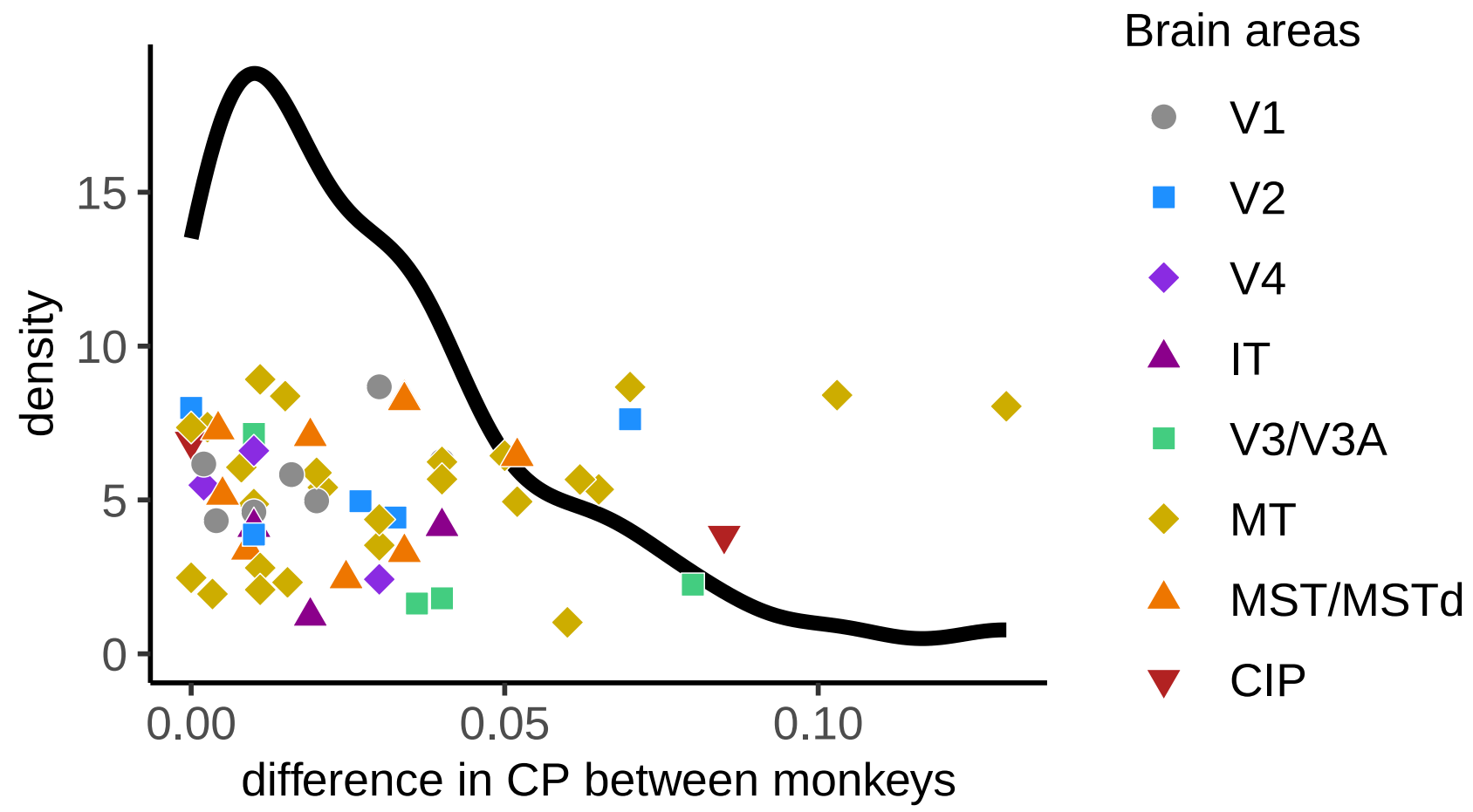

Figure S2: **Differences in mean CP between monkeys within the same study.** Each point shows the difference between two monkeys, computed by subtracting the lower CP value from the higher. Vertical jitter was added for visualization. The black curve is a density function

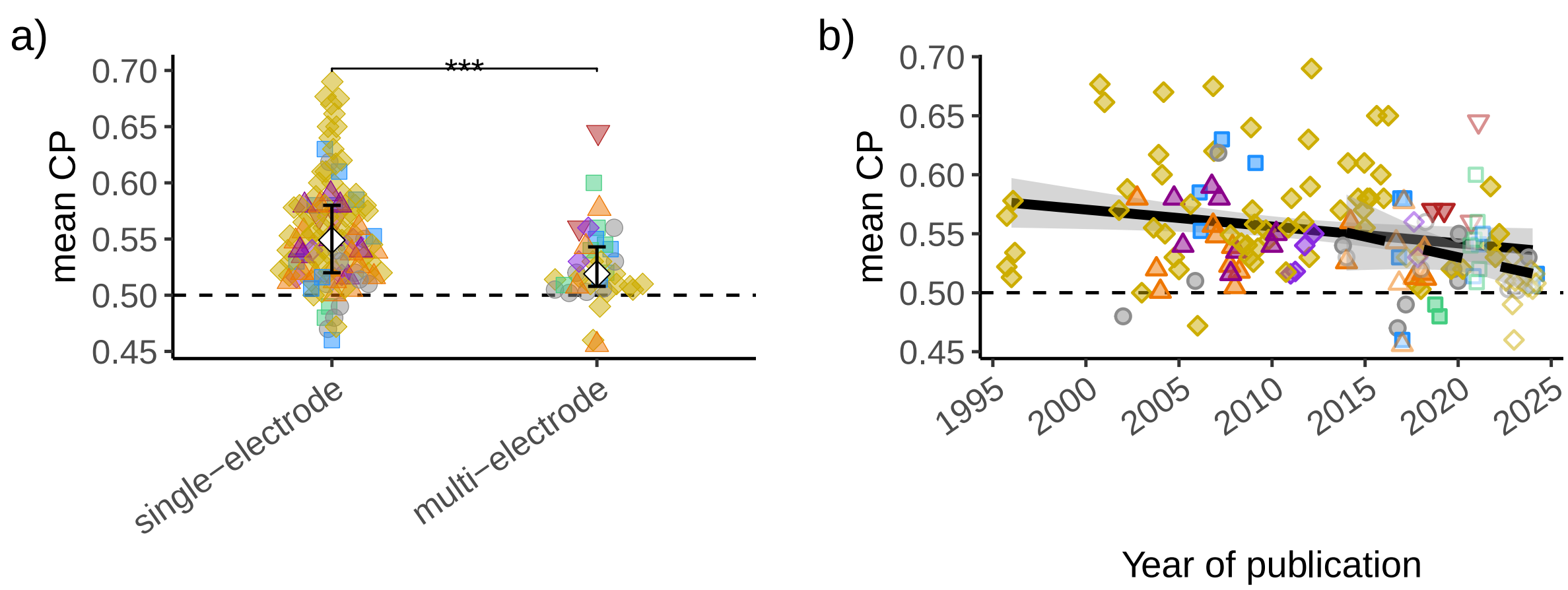

Figure S3: **Mean CP is lower in multi-electrode studies and declines over time in single-electrode studies. (a)** Mean CP by recording technique. Multi-electrode studies report significantly lower CP values (*** $p < 0.001$). **(b)** Relationship between mean CP and year of publication, shown separately for single-electrode (filled symbols, solid regression line) and multi-electrode studies (open symbols, dashed line). A significant decline is observed even when restricting the analysis to single-electrode studies ($\beta = -0.0015$, $p = 0.02$).

bioRxiv preprint doi: https://doi.org/10.64898/2026.06.08.730961; this version posted June 11, 2026. The copyright holder for this preprint (which was not certified by peer review) is the author/funder, who has granted bioRxiv a license to display the preprint in perpetuity. It is made available under a CC-BY 4.0 International license.

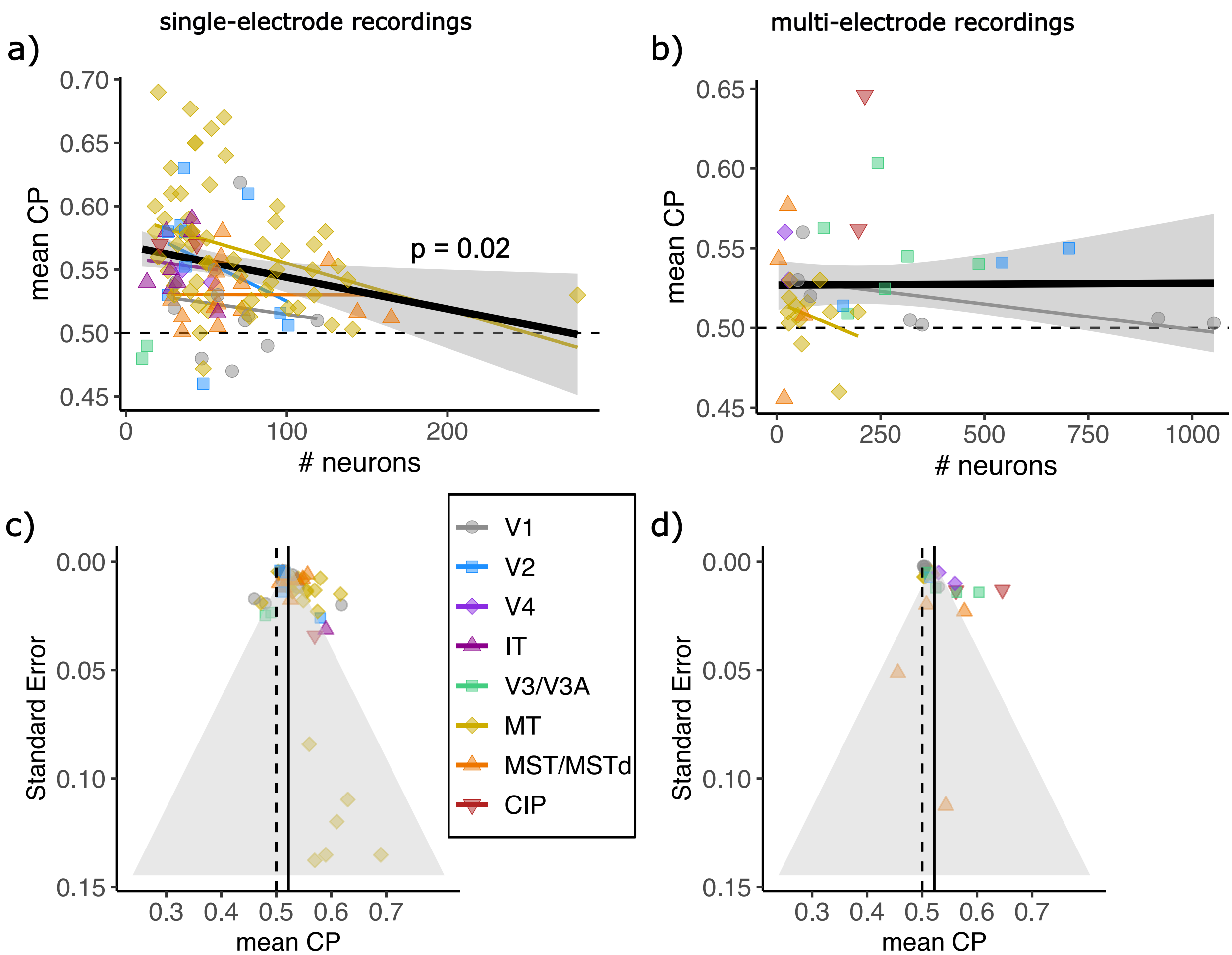

Figure S4: **Mean CP values decrease with statistical power of the study. (a)** Relationship between mean CP and the number of neurons contributing to the estimate, restricted to single-electrode studies to reduce confounding. A negative trend (slope = -0.0003, $p = 0.02$, $N = 109$) suggests that low-powered studies (fewer neurons) tend to report inflated CP values. **(b)** same as (a) but restricted to multi-electrode studies. No trend is observed ($p = 0.96$, $N = 37$) **(c)** Funnel plot showing standard error (y-axis) versus mean CP (x-axis). Restricted to single-electrode studies to reduce confounding. The solid vertical line marks the meta-analytic mean CP (0.52), computed as an inverse-variance-weighted average. The shaded triangle represents the 95% expected region assuming this true mean. An asymmetry is evident, with higher-error studies skewed toward elevated CP values, consistent with publication bias (Egger's test: $p = 0.01$, $N = 51$). **(d)** same as (c) but restricted to multi-electrode studies. Similar asymmetry as for single-electrode studies is observed (Egger's test: $p < 0.001$, $N = 24$).

bioRxiv preprint doi: https://doi.org/10.64898/2026.06.08.730961; this version posted June 11, 2026. The copyright holder for this preprint (which was not certified by peer review) is the author/funder, who has granted bioRxiv a license to display the preprint in perpetuity. It is made available under a CC-BY 4.0 International license.

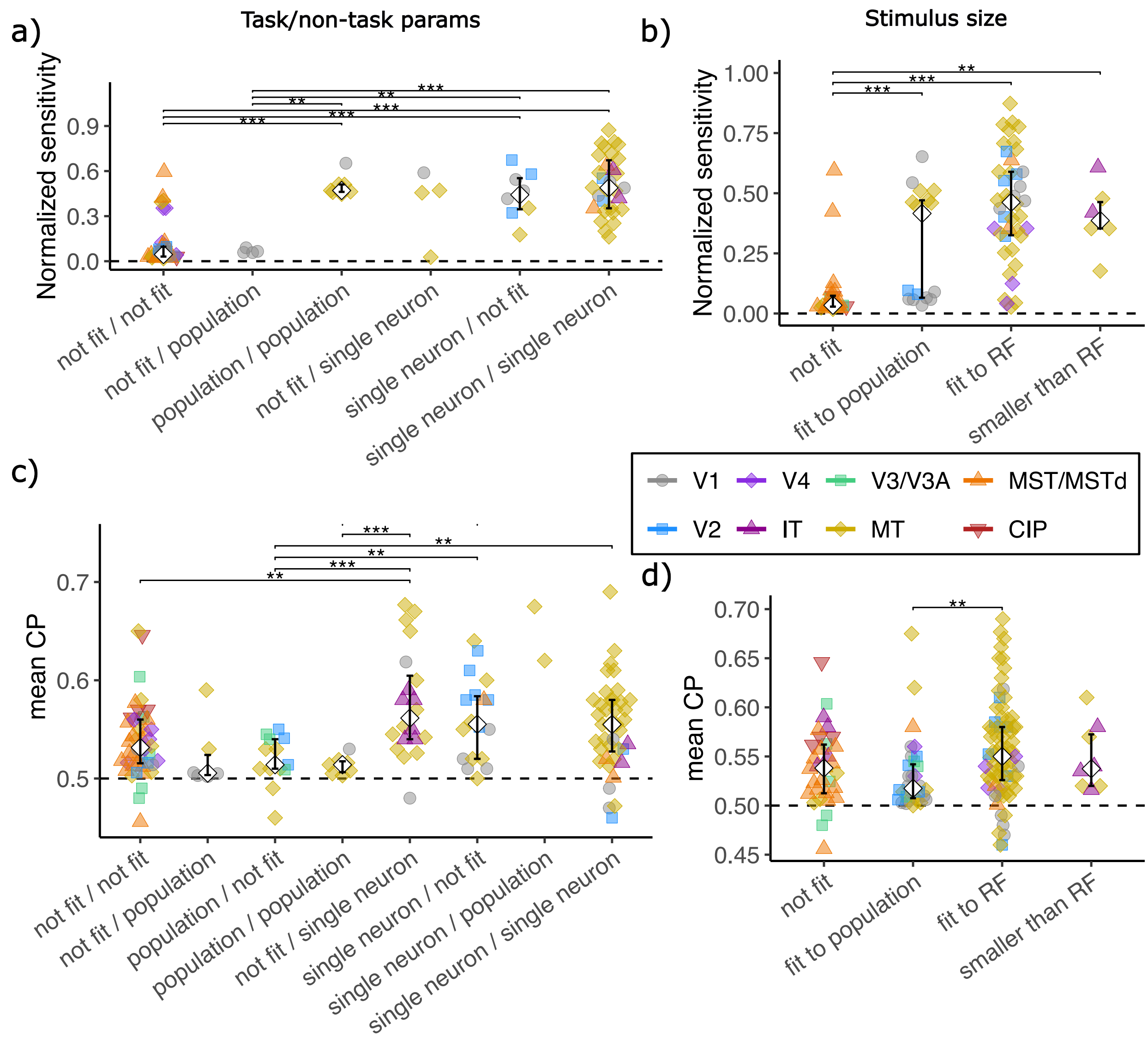

Figure S5: **Effect of tailoring techniques on neuronal sensitivity (a, b) and mean CP (c, d).** Left column (**a, c**) shows the effect of tailoring two types of stimulus parameters: task-relevant ("task") and task-irrelevant but response-modulating ("non-task"). X-axis labels represent combinations of tailoring choices. For example, the label 'not fit/single neuron' indicates that the task parameter was not tailored, whereas the non-task parameter was optimized for each individual neuron. Note that in panel **a**, nearly all available sensitivity data come from single-electrode studies, so tailoring to population-level properties is mostly absent. Right column (**b, d**) shows the effect of tailoring stimulus size relative to the receptive field. Asterisks indicate significant differences between two categories based on Mann–Whitney tests (* $p < 0.05$, ** $p < 0.01$, *** $p < 0.001$). For **a, c** $p < 0.05$ results are omitted as they would not survive correction for multiple comparisons.

bioRxiv preprint doi: https://doi.org/10.64898/2026.06.08.730961; this version posted June 11, 2026. The copyright holder for this preprint (which was not certified by peer review) is the author/funder, who has granted bioRxiv a license to display the preprint in perpetuity. It is made available under a CC-BY 4.0 International license.

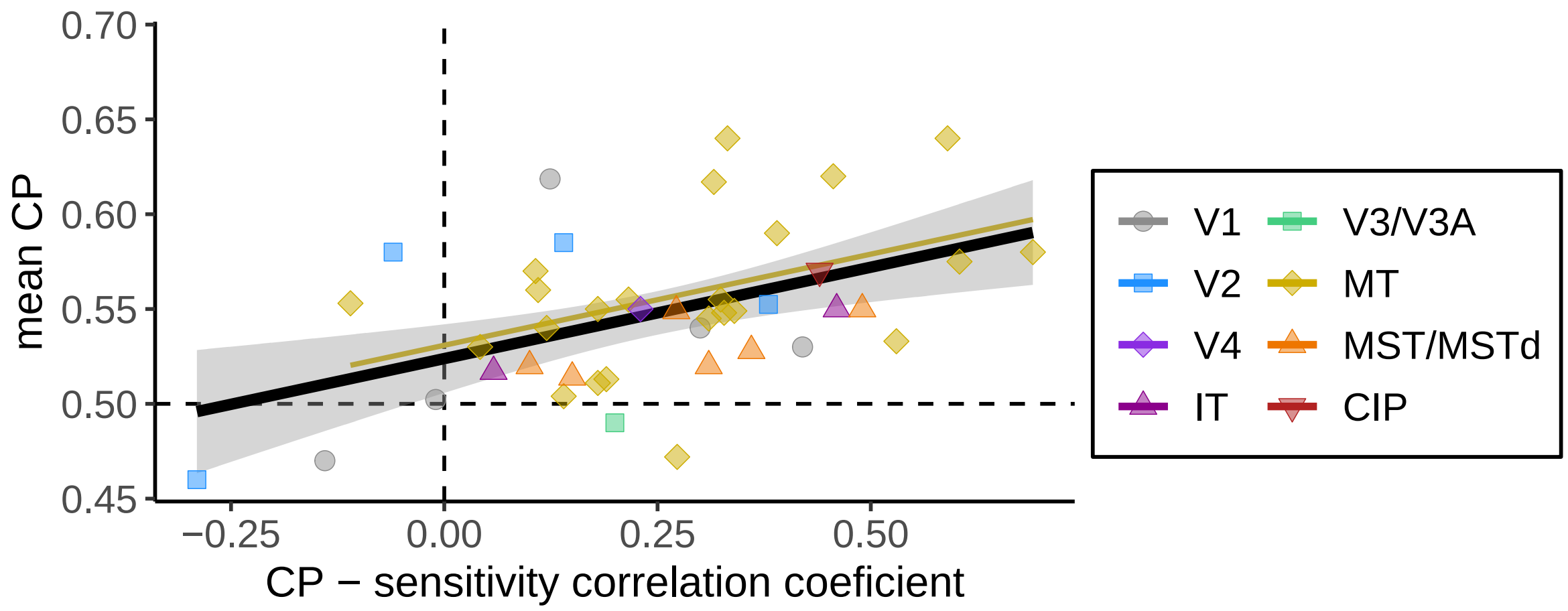

Figure S6: **Relationship between mean CP and the within study CP–sensitivity correlation coefficient (Pearson or Spearman).** For comparability, coefficients based on neuronal thresholds were sign-inverted. The black line shows the overall regression (slope = 0.10, $p = 0.001$, N = 41); the thin golden line shows the regression within MT (data from other areas were too sparse for separate analysis).

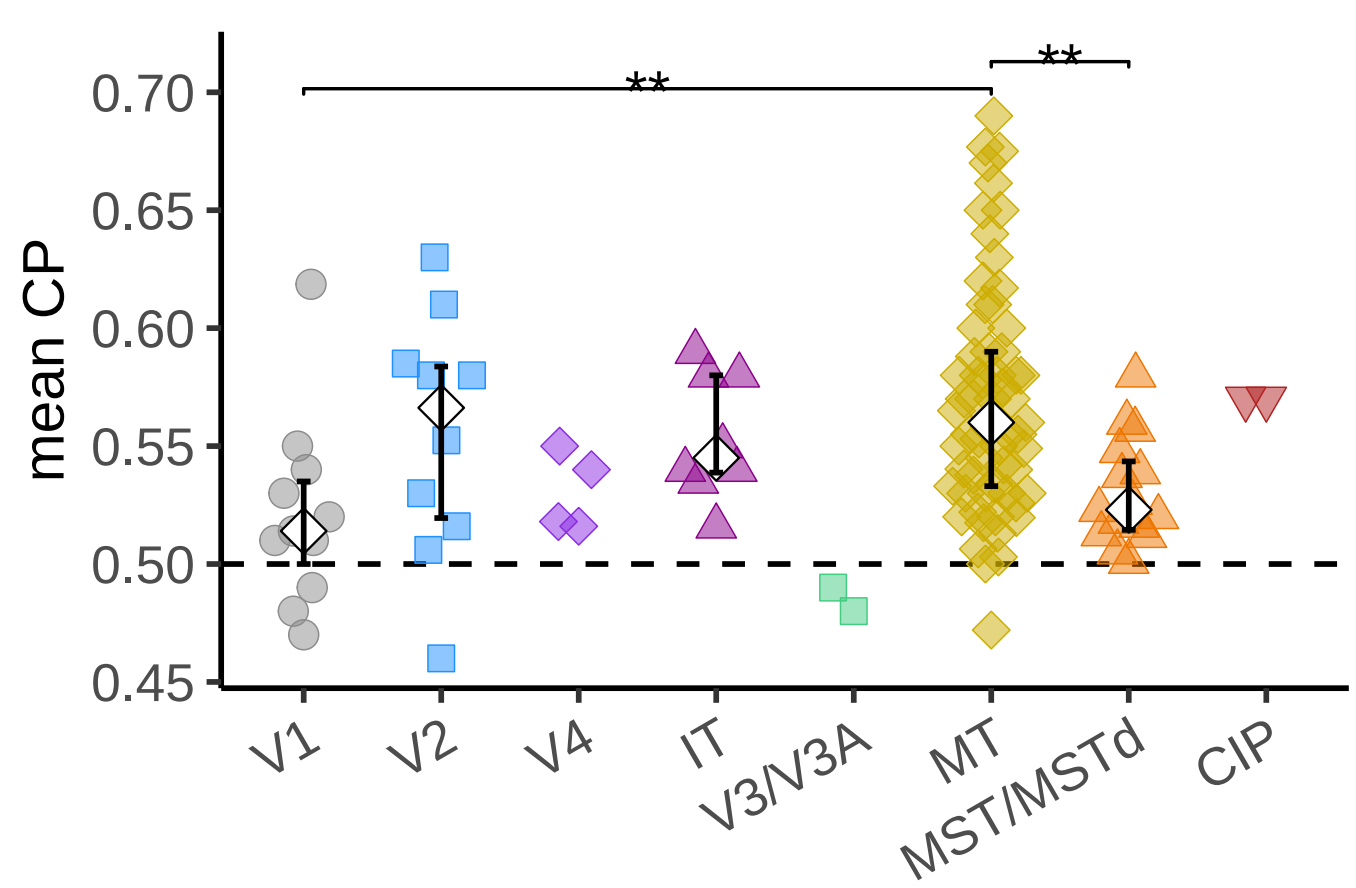

Figure S7: **Mean CP values across brain areas for single-electrode recordings** Diamonds and error bars represent medians and interquartile ranges for areas with more than five observations. Asterisks indicate significant differences between two brain areas based on Mann–Whitney tests (** $p < 0.01$, *** $p < 0.001$); $p < 0.05$ results are omitted as they would not survive correction for multiple comparisons.

bioRxiv preprint doi: https://doi.org/10.64898/2026.06.08.730961; this version posted June 11, 2026. The copyright holder for this preprint (which was not certified by peer review) is the author/funder, who has granted bioRxiv a license to display the preprint in perpetuity. It is made available under a CC-BY 4.0 International license.

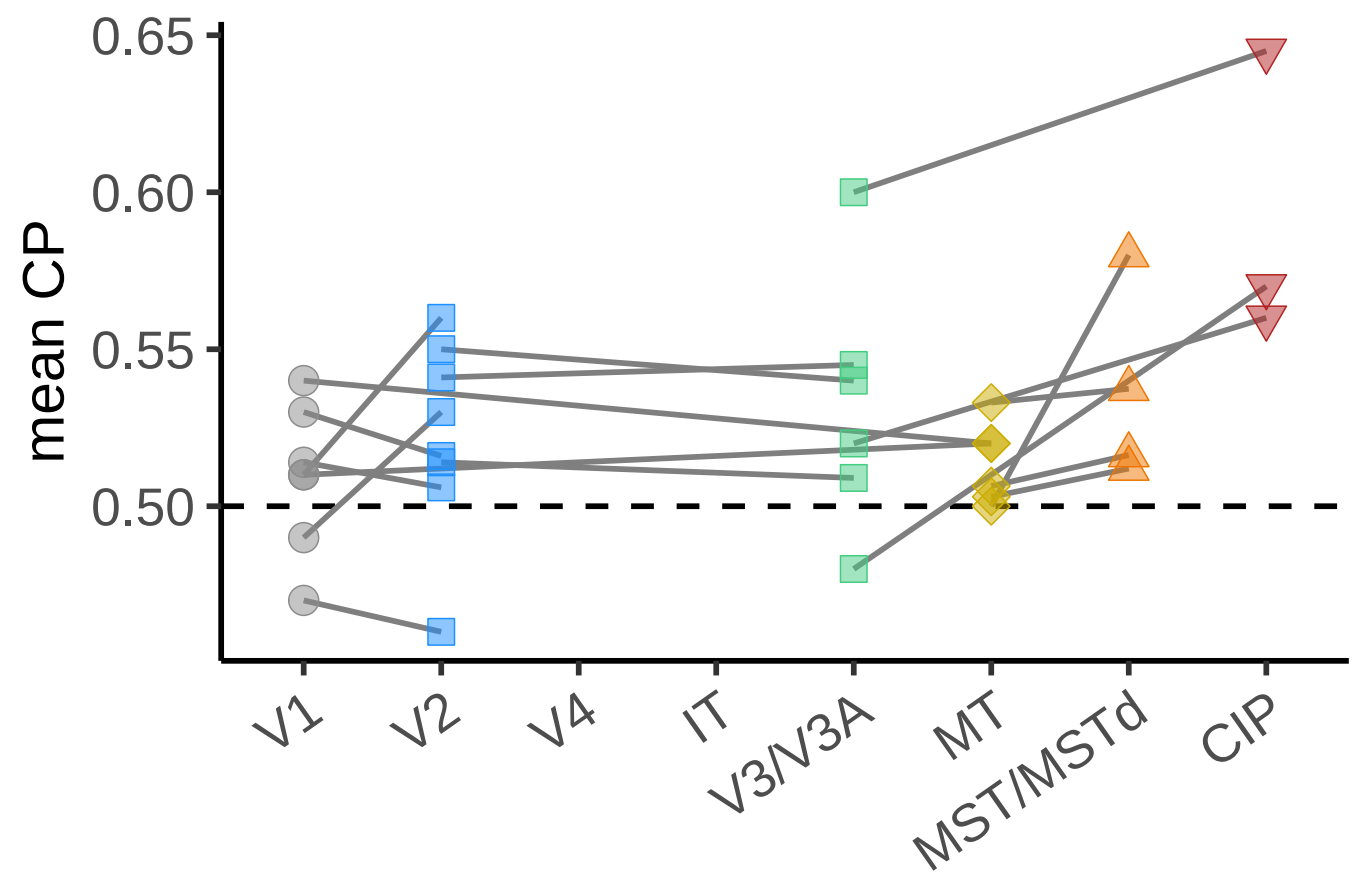

Figure S8: **Mean CP values across brain areas within the same study and monkey.** Lines connect data points from the same monkey within each study.

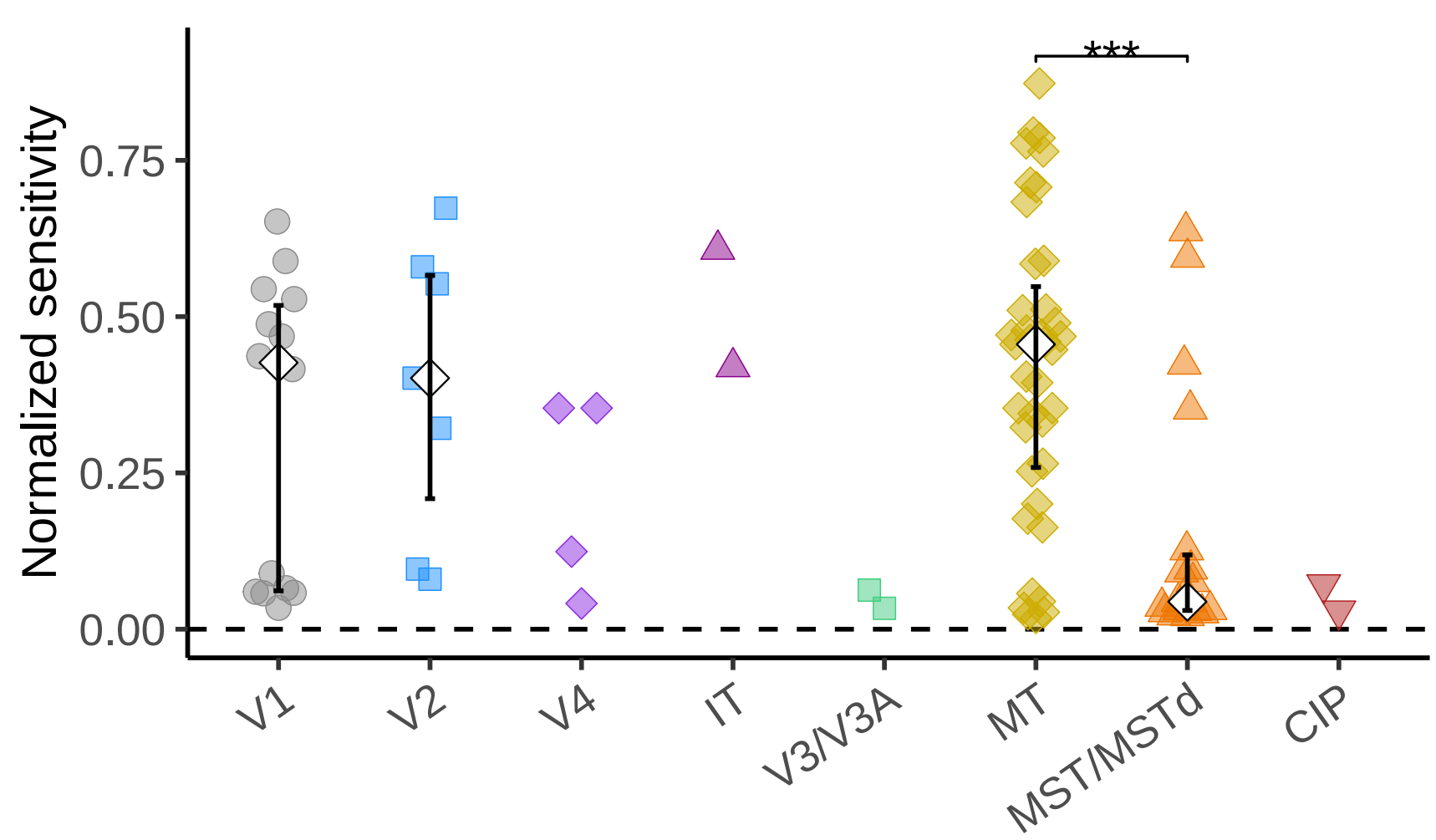

Figure S9: **Comparison of task sensitivities between brain areas** Asterisks indicate significant differences between two brain areas based on Mann–Whitney tests (** $p < 0.01$, *** $p < 0.001$); $p < 0.05$ results are omitted as they would not survive correction for multiple comparisons.

bioRxiv preprint doi: https://doi.org/10.64898/2026.06.08.730961; this version posted June 11, 2026. The copyright holder for this preprint (which was not certified by peer review) is the author/funder, who has granted bioRxiv a license to display the preprint in perpetuity. It is made available under a CC-BY 4.0 International license.

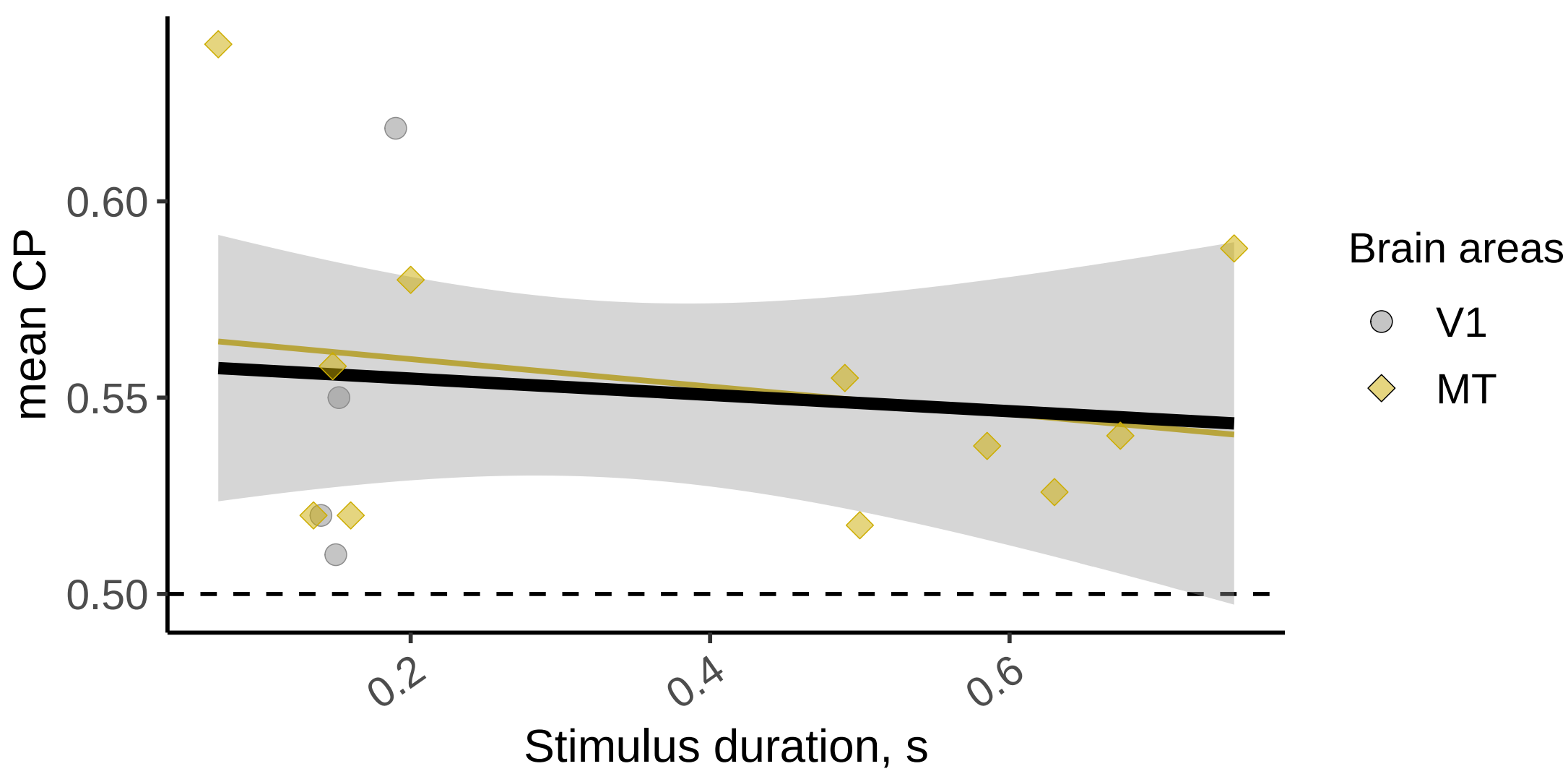

Figure S10: **Relationship between mean CP and stimulus duration for reaction time tasks**

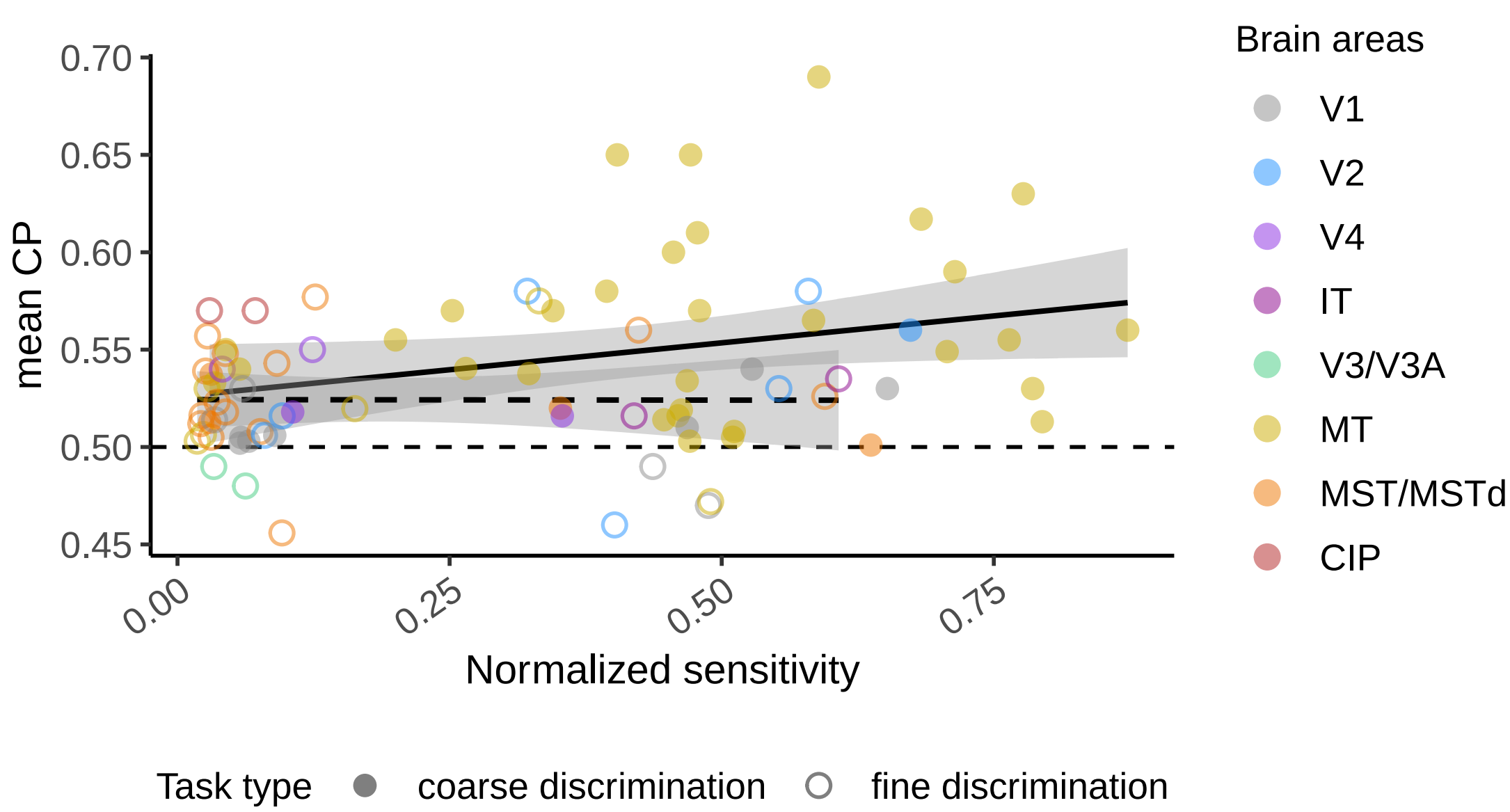

Figure S11: **Relationship between mean CP and normalized sensitivity for fine- vs. coarse-discrimination tasks.** Linear regression analyses were performed separately for the two task types: solid line – coarse- and dashed line – fine-discrimination task). A separate model with interaction terms revealed no significant difference in slope or intercept: $\Delta\beta = \beta_{\text{fine}} - \beta_{\text{coarse}} = -0.03$, $p = 0.3$; $\Delta\beta_0 = -0.007$, $p = 0.7$.

bioRxiv preprint doi: https://doi.org/10.64898/2026.06.08.730961; this version posted June 11, 2026. The copyright holder for this preprint (which was not certified by peer review) is the author/funder, who has granted bioRxiv a license to display the preprint in perpetuity. It is made available under a CC-BY 4.0 International license.

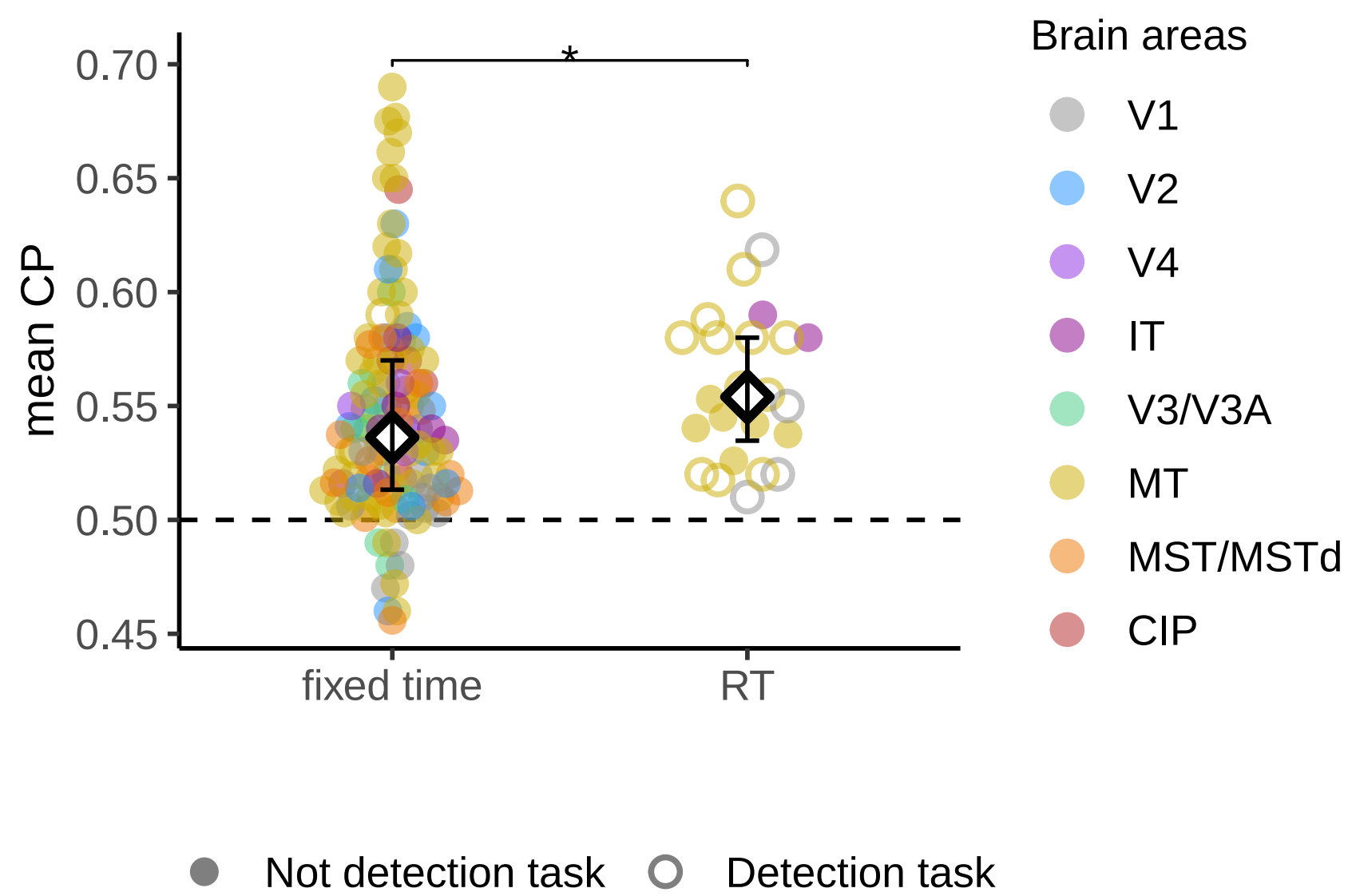

Figure S12: **Relationship between Mean CP values with whether experiment have reaction-time design.** The empty circles represent detection tasks; solid circles – all the other task types. In RT tasks (N = 24), 16 data points are from the detection tasks, 8 – from coarse-discrimination ones.

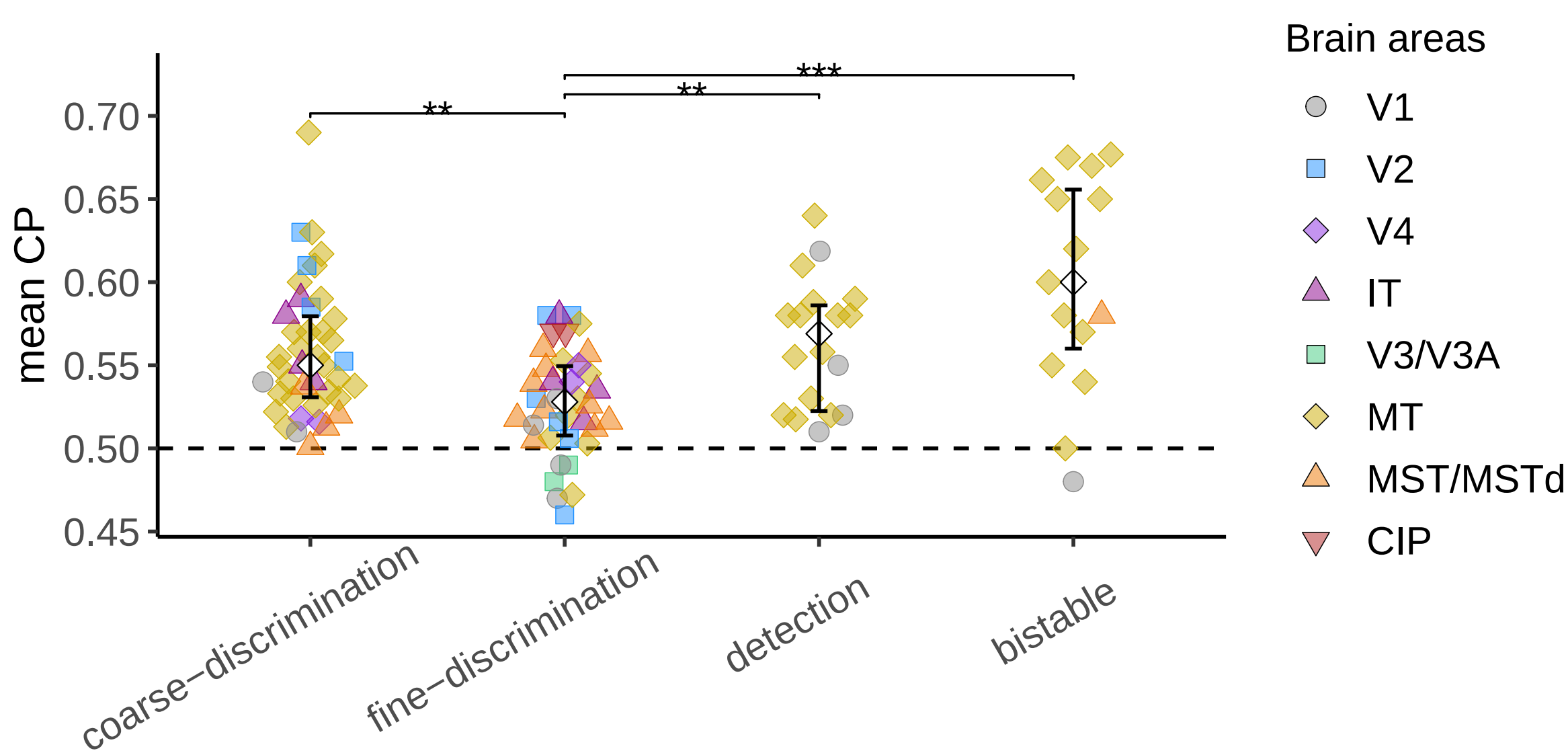

Figure S13: **Mean CP values across task types for single-electrode recordings** Diamonds and error bars represent medians and interquartile ranges. Asterisks indicate significant differences between two task types based on Mann–Whitney tests (** $p < 0.01$, *** $p < 0.001$).

bioRxiv preprint doi: https://doi.org/10.64898/2026.06.08.730961; this version posted June 11, 2026. The copyright holder for this preprint (which was not certified by peer review) is the author/funder, who has granted bioRxiv a license to display the preprint in perpetuity. It is made available under a CC-BY 4.0 International license.

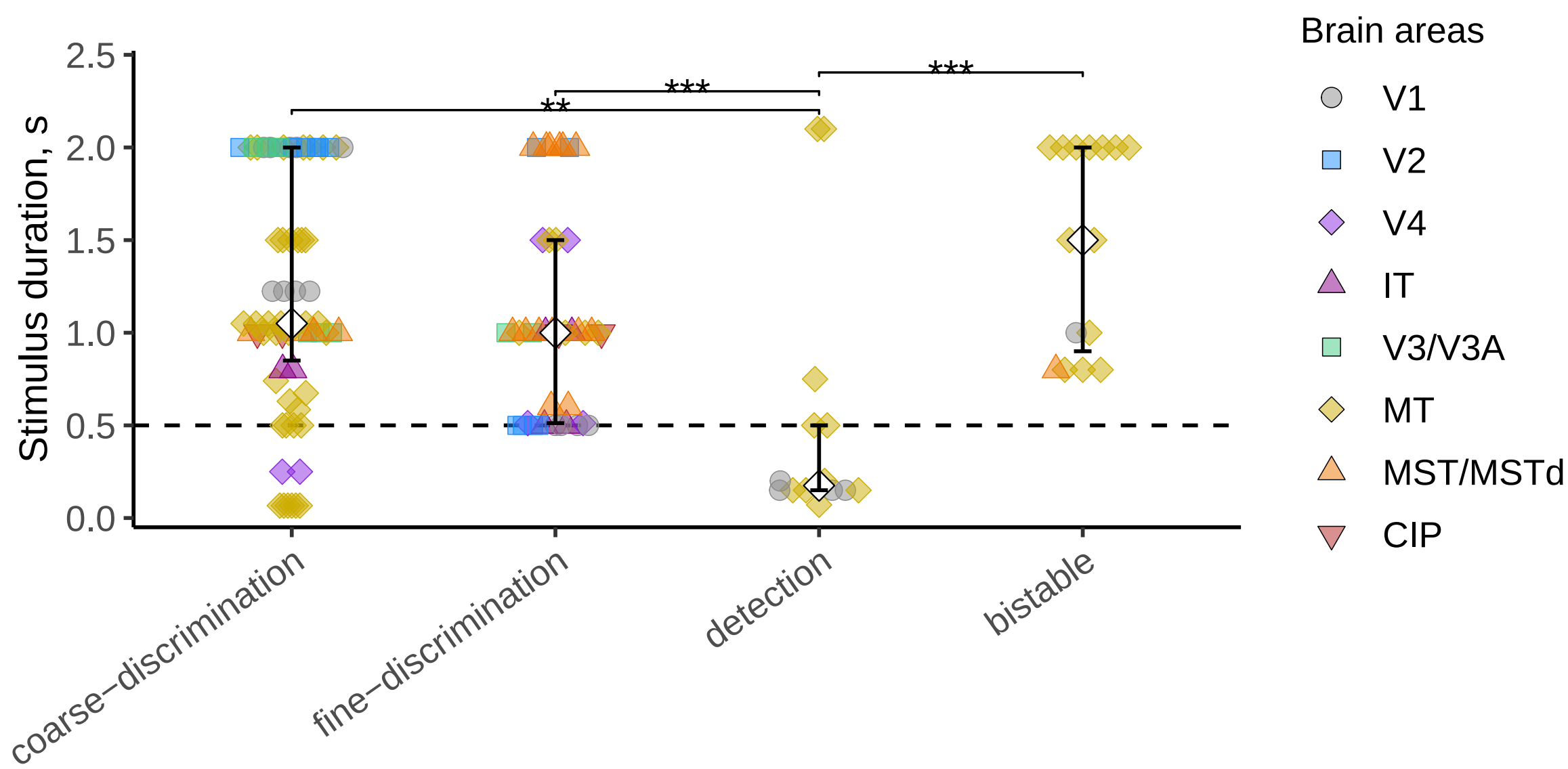

Figure S14: **Relationship between stimulus duration and task type.** Asterisks indicate significant differences between two task types based on Mann–Whitney tests (** $p < 0.01$, *** $p < 0.001$).

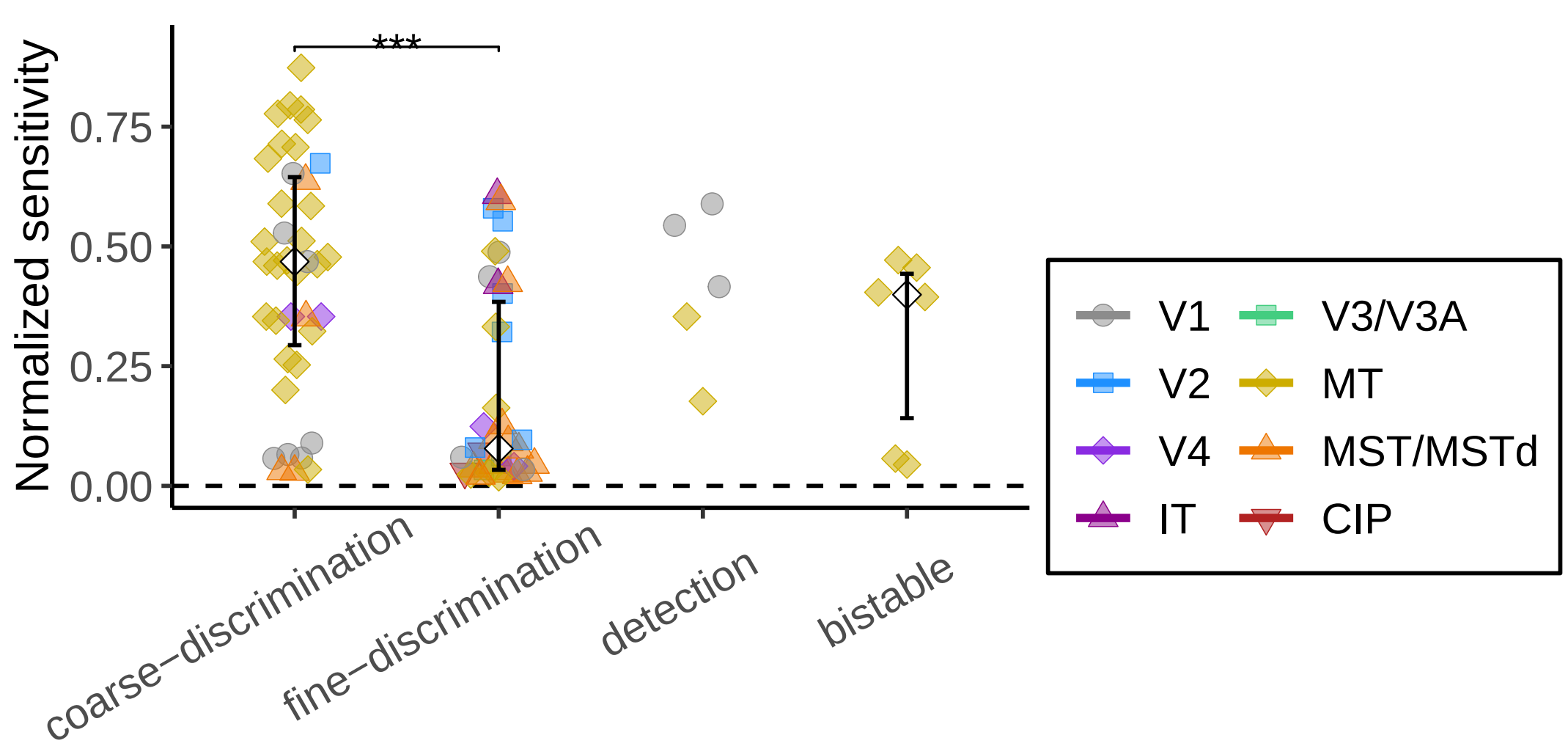

Figure S15: **Fine-discrimination tasks show substantially lower sensitivity, which may account for their reduced mean CP.** Normalized neuronal sensitivity (inverse mean N/P ratio) across task types. Diamonds and error bars represent medians and interquartile ranges with more than five observations. Asterisks indicate significant differences between two task types based on Mann–Whitney tests (** $p < 0.01$, *** $p < 0.001$).

bioRxiv preprint doi: https://doi.org/10.64898/2026.06.08.730961; this version posted June 11, 2026. The copyright holder for this preprint (which was not certified by peer review) is the author/funder, who has granted bioRxiv a license to display the preprint in perpetuity. It is made available under a CC-BY 4.0 International license.

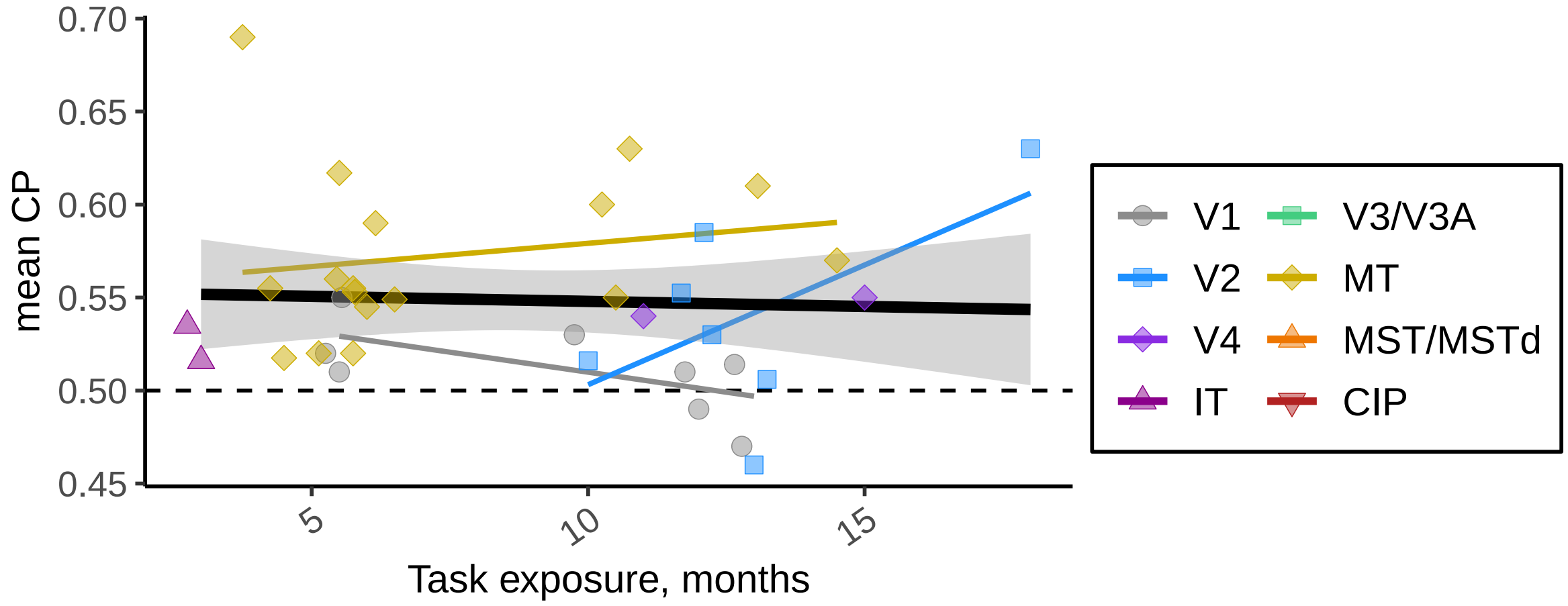

Figure S16: **Relationship between mean CP values and task exposure.**

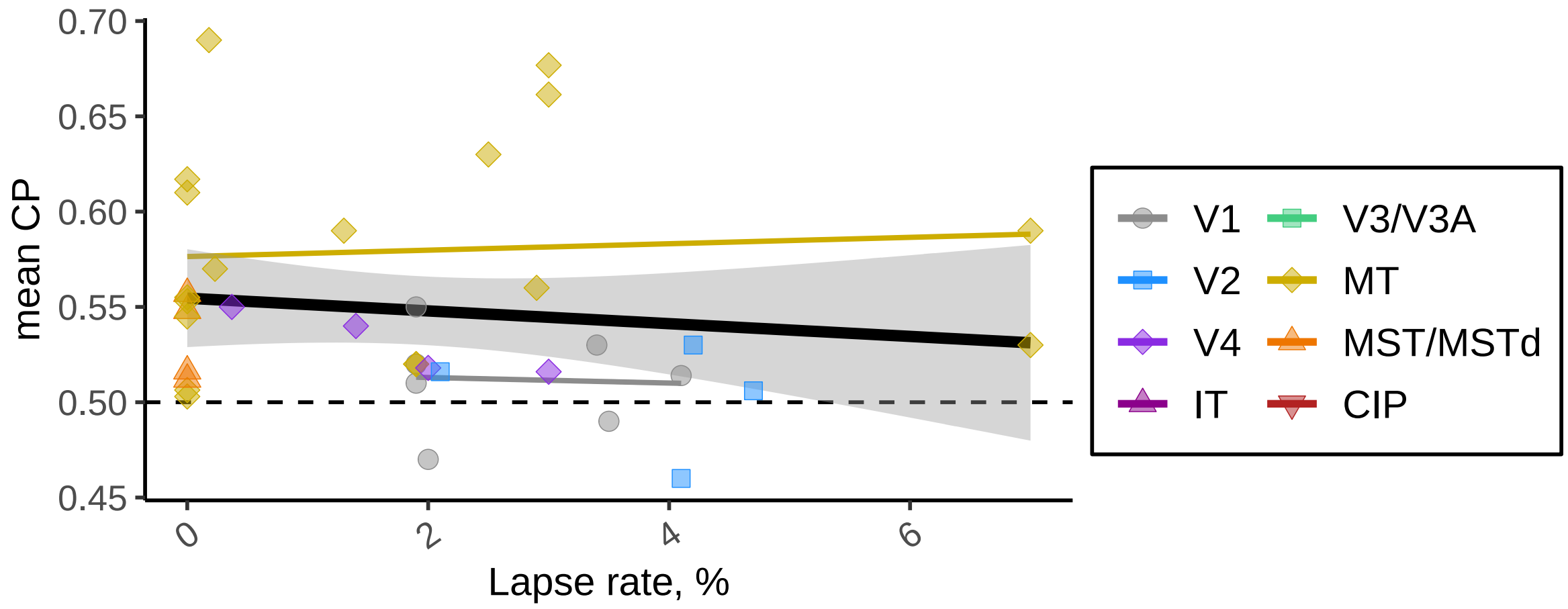

Figure S17: **Relationship between Mean CP values and monkey's lapse rate.**

bioRxiv preprint doi: https://doi.org/10.64898/2026.06.08.730961; this version posted June 11, 2026. The copyright holder for this preprint (which was not certified by peer review) is the author/funder, who has granted bioRxiv a license to display the preprint in perpetuity. It is made available under a CC-BY 4.0 International license.

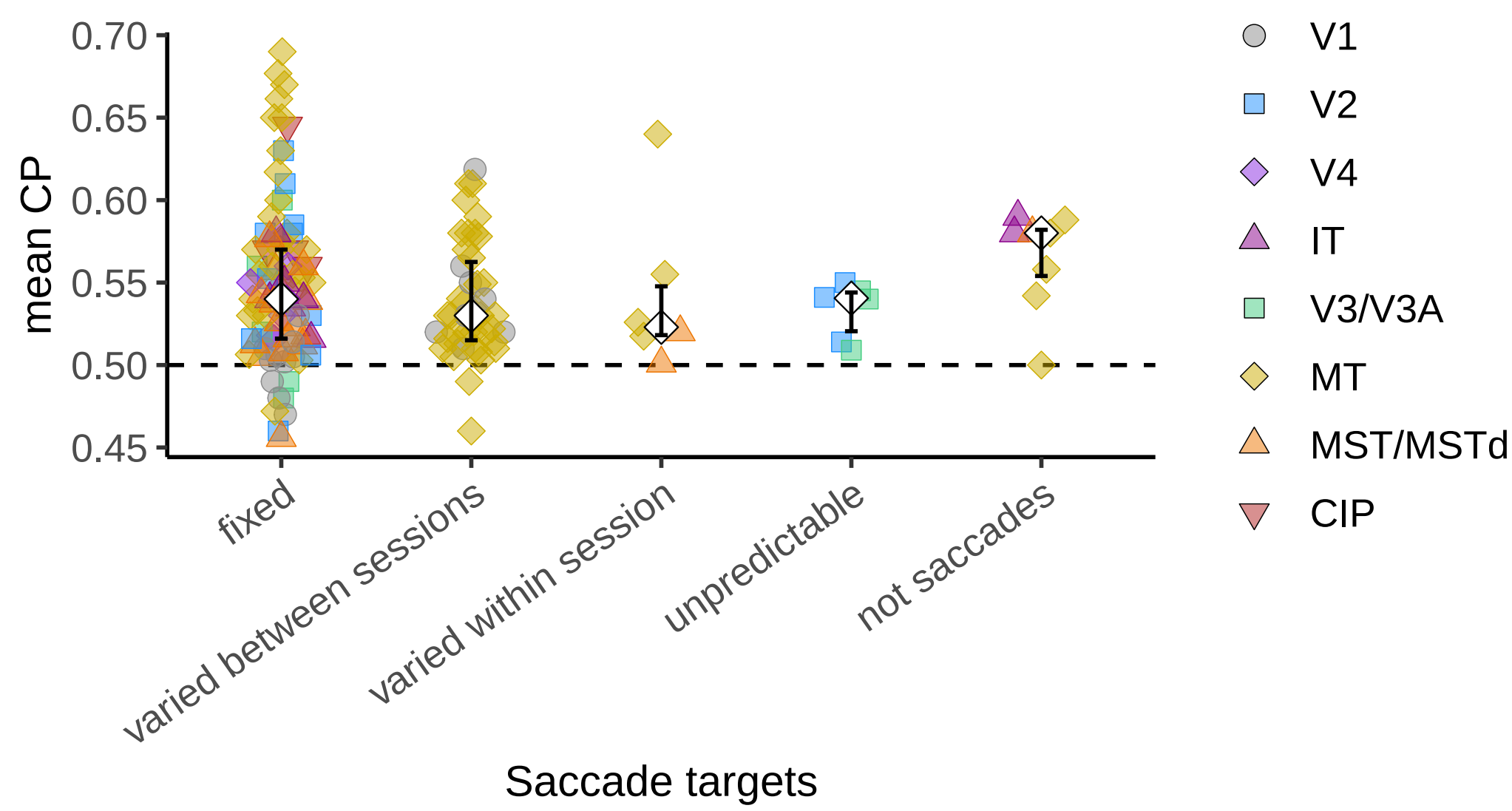

Figure S18: **Relationship between Mean CP values and predictability of targets**. No pairwise comparisons were statistically significant (Mann-Whitney test, corrected for multiple comparisons).

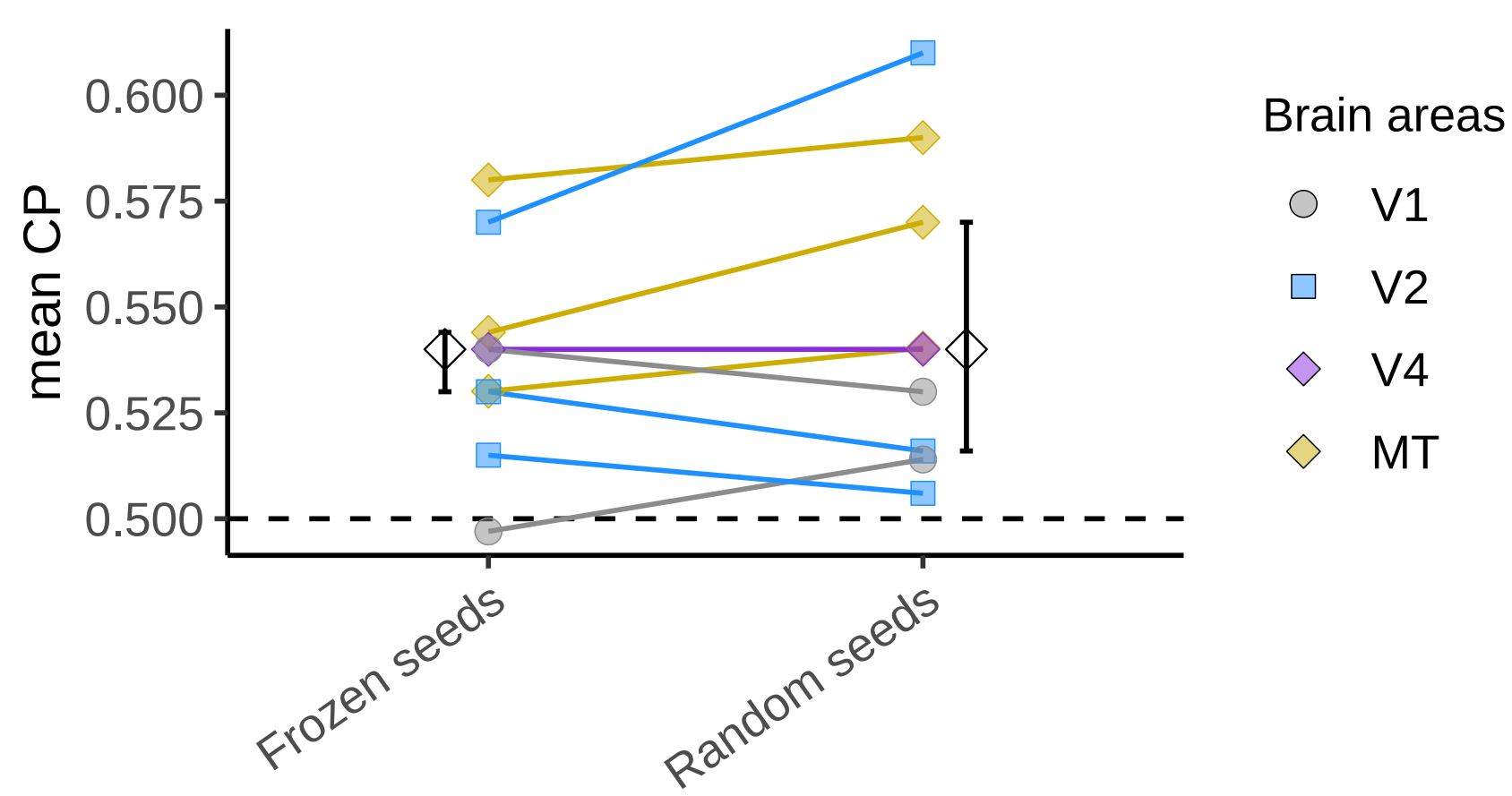

Figure S19: **Relationship between mean CP values and stimulus noise structure.** Mean CP values are shown for conditions using frozen vs. random stimulus seeds. Lines connect data points from the same study. Wilcoxon signed-rank tests have not revealed statistical difference ($p = 0.3$).

bioRxiv preprint doi: https://doi.org/10.64898/2026.06.08.730961; this version posted June 11, 2026. The copyright holder for this preprint (which was not certified by peer review) is the author/funder, who has granted bioRxiv a license to display the preprint in perpetuity. It is made available under a CC-BY 4.0 International license.

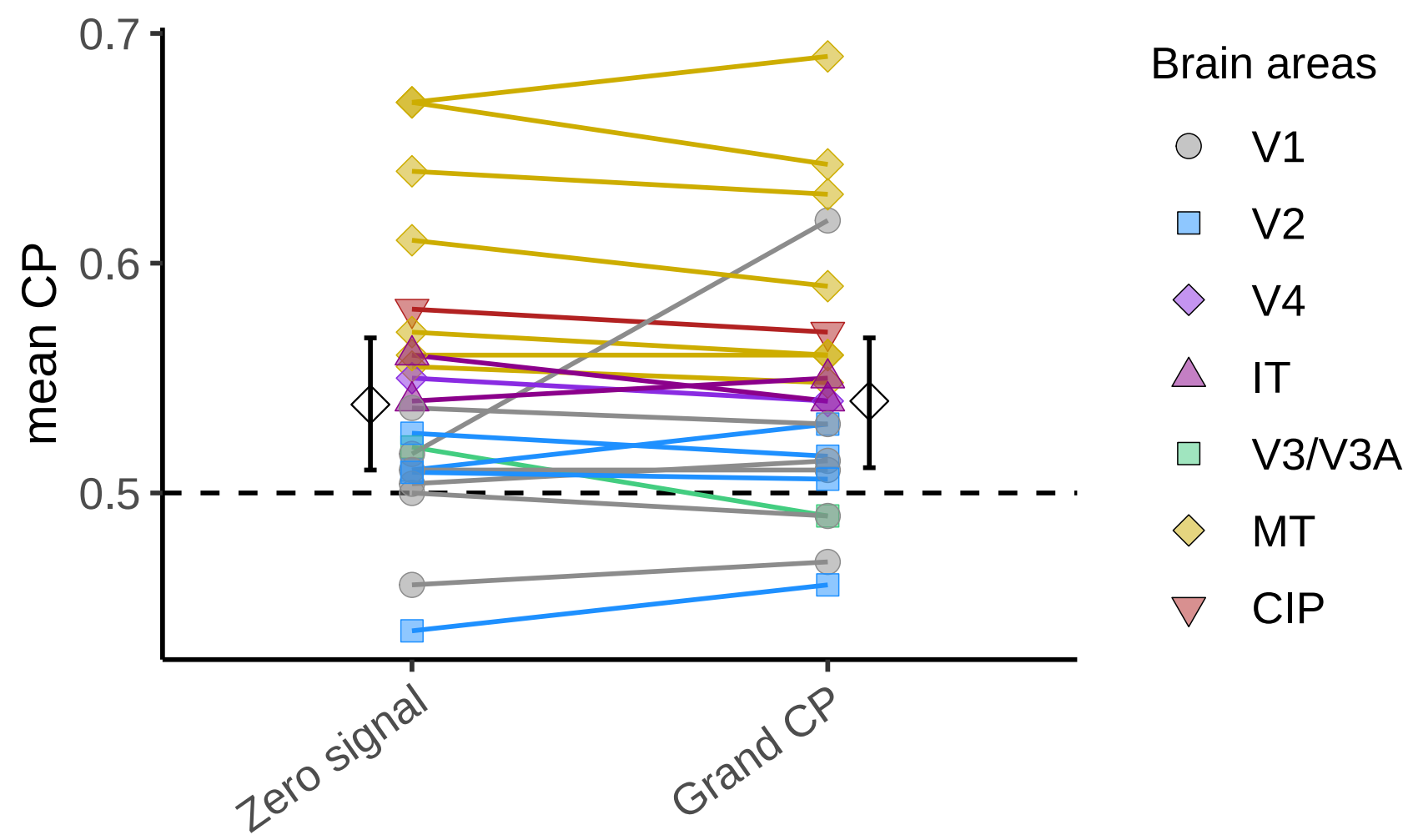

Figure S20: **Relationship between mean CP values and CP estimation method**. Lines connect data points from the same study. Wilcoxon signed-rank test has not revealed statistical difference ($p = 0.4$)

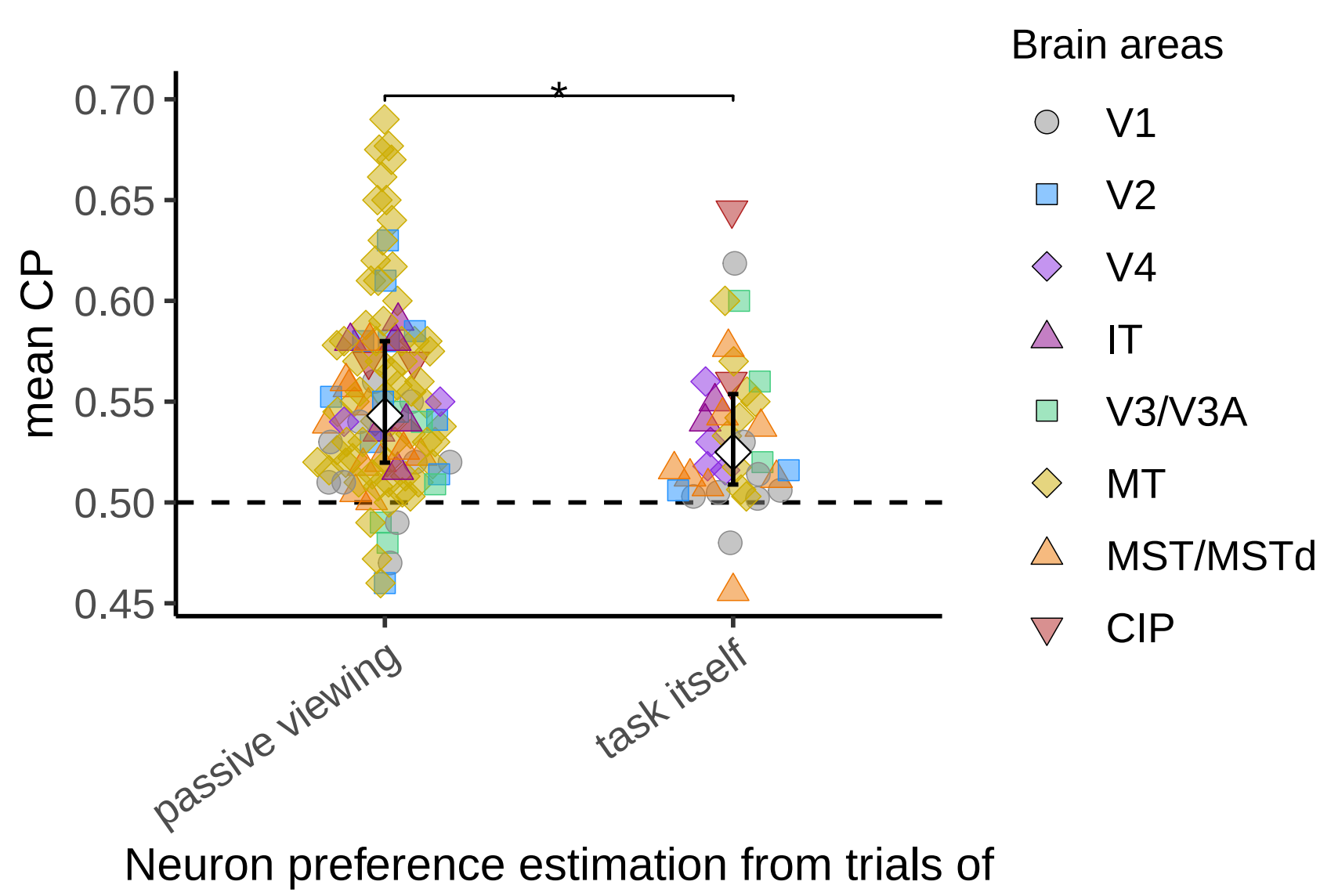

Figure S21: **Relationship between Mean CP values and method of neuron preference estimation**. Mann–Whitney test revealed that studies with preferences estimated from the task trials exhibited lower mean CP compared to those that used preferences estimated from passive viewing sessions (* asterisk in the figure, $\beta = -0.018$, $p = 0.03$), but this effect appears to be driven by other factors (see section 8.3).

bioRxiv preprint doi: https://doi.org/10.64898/2026.06.08.730961; this version posted June 11, 2026. The copyright holder for this preprint (which was not certified by peer review) is the author/funder, who has granted bioRxiv a license to display the preprint in perpetuity. It is made available under a CC-BY 4.0 International license.

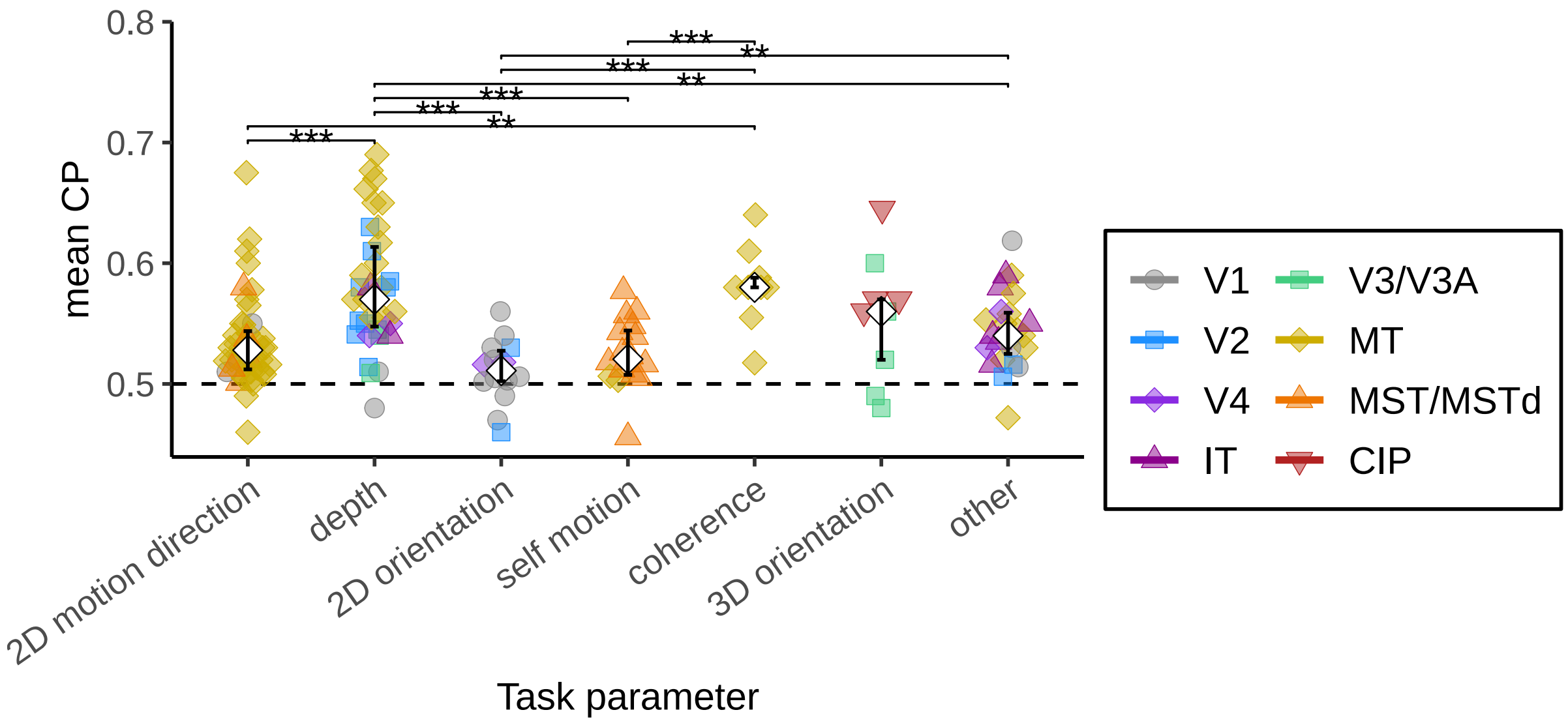

Figure S22: **Relationship between Mean CP values and task variable**. Asterisks indicate significant differences between two task variables based on Mann–Whitney tests (** $p < 0.01$, *** $p < 0.001$); $p < 0.05$ results are omitted as they would not survive correction for multiple comparisons. Note that the observed difference is largely driven by the effect of brain area, as task characteristics and brain area are strongly coupled.

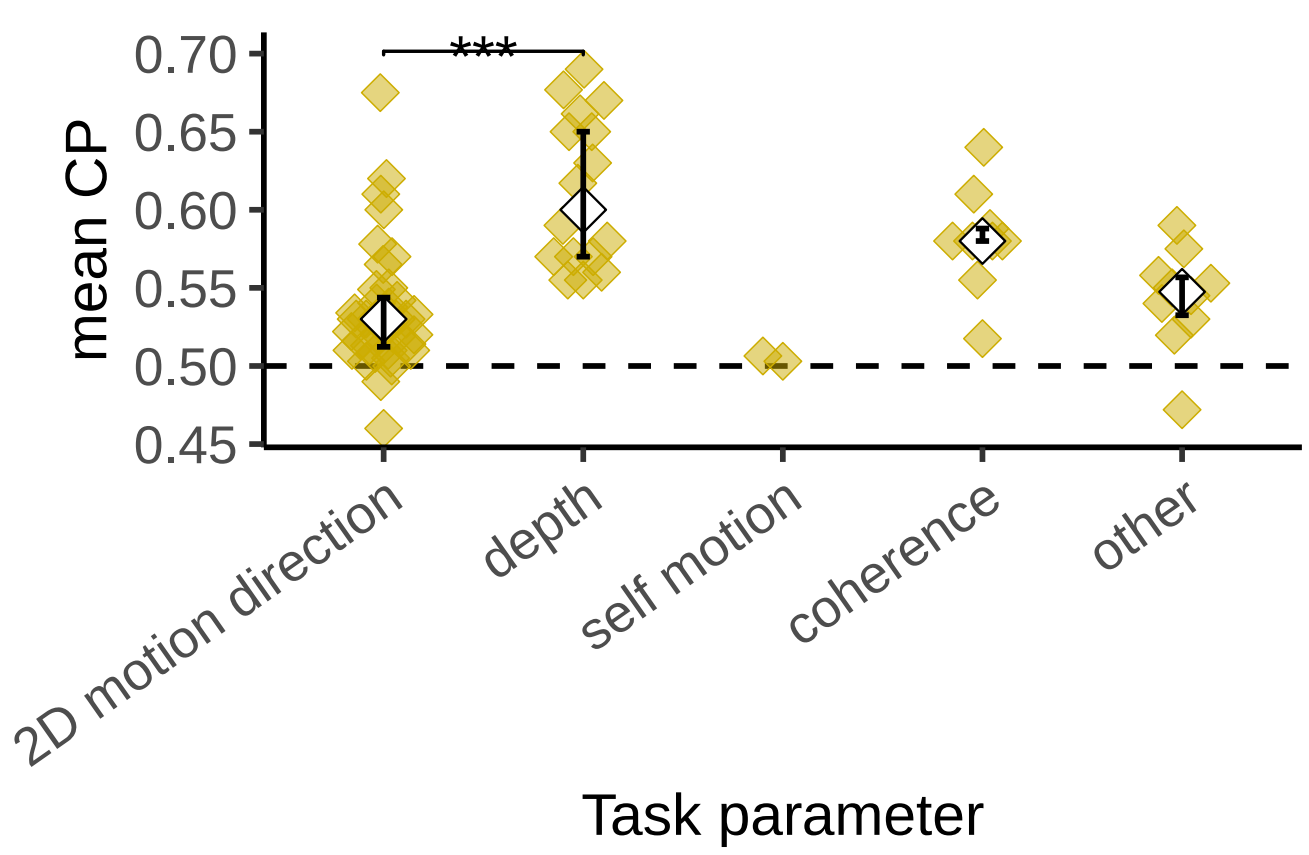

Figure S23: **Relationship between mean CP values and task variable within area MT.** Asterisks indicate significant differences between task conditions based on Mann–Whitney tests (*** $p < 0.001$); results with $p < 0.05$ are omitted as they would not survive correction for multiple comparisons. Unlike the across-area analysis, the observed difference between motion and depth discrimination tasks in MT cannot be attributed to brain area and remains significant after controlling for sensitivity, stimulus duration, and task type (see section 8.3).

bioRxiv preprint doi: https://doi.org/10.64898/2026.06.08.730961; this version posted June 11, 2026. The copyright holder for this preprint (which was not certified by peer review) is the author/funder, who has granted bioRxiv a license to display the preprint in perpetuity. It is made available under a CC-BY 4.0 International license.

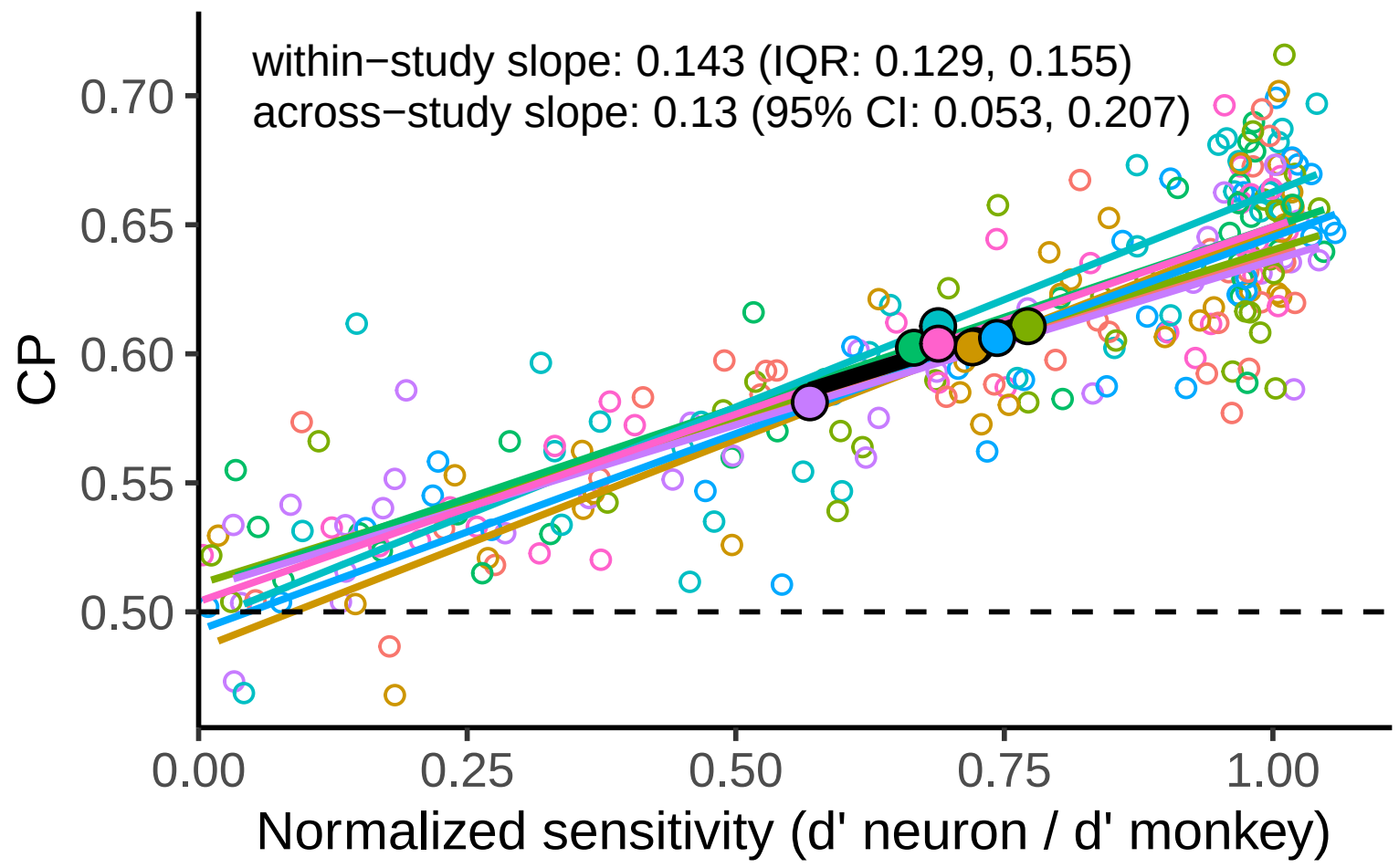

Figure S24: **Hierarchical inference model simulations with task-aligned feedback and incomplete learning predict within- and across-study CP–normalized sensitivity slopes of approximately 0.15.** Normalized sensitivity (x-axis) is defined as the ratio $d'_{\text{neuron}}/d'_{\text{behavior}}$, where d'_{neuron} is estimated during task performance and reflect both feedforward and feedback influences (see Section 2.4.1). Simulations utilized the hierarchical inference model of Haefner et al. (2016) with 256 sensory neurons randomly divided into 8 "studies" of 32 neurons each (indicated by color). Feedback strength was controlled by the parameter δ and set to 0.08—the maximum value used in Haefner et al. (2016)—which corresponds to proficient but incomplete task learning. Small open circles represent individual neurons, and large filled circles denote study means. Thin lines indicate within-study regressions; the thick black line represents the across-study regression.

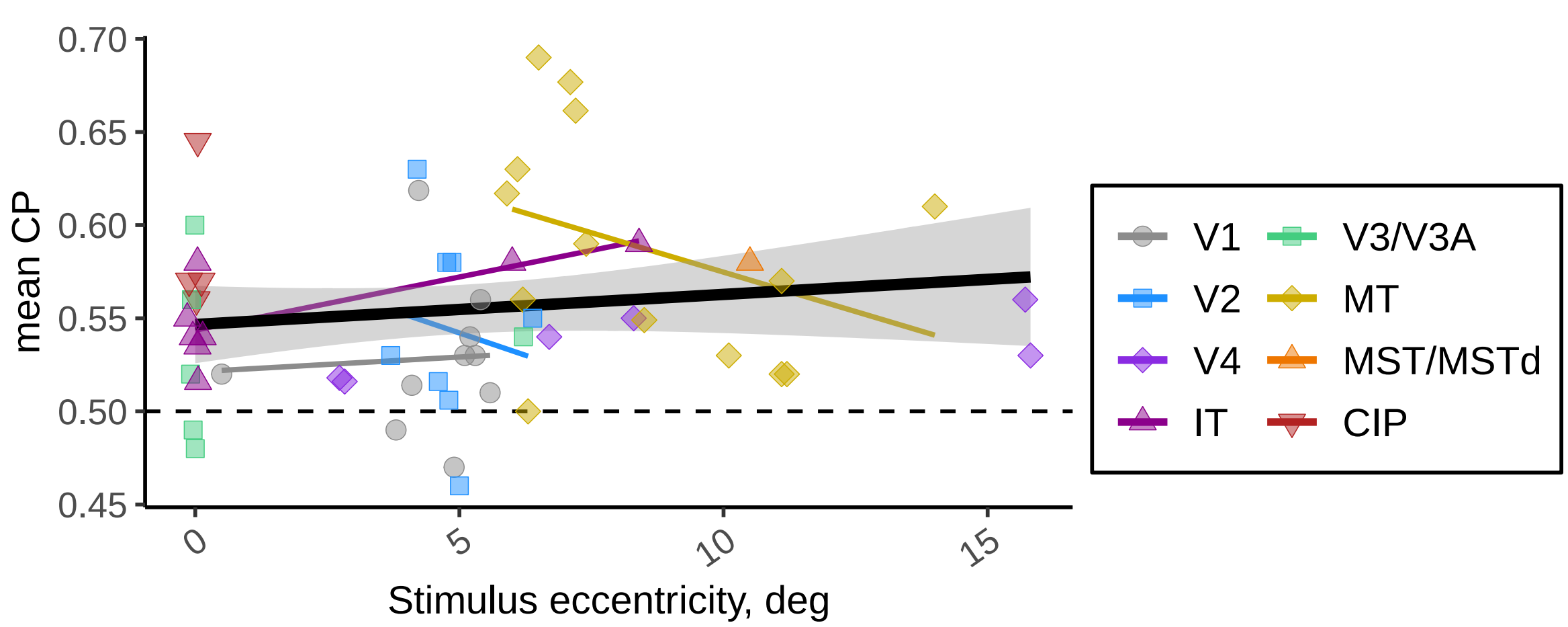

Figure S25: **Relationship between mean CP values and stimulus eccentricity.**

bioRxiv preprint doi: https://doi.org/10.64898/2026.06.08.730961; this version posted June 11, 2026. The copyright holder for this preprint (which was not certified by peer review) is the author/funder, who has granted bioRxiv a license to display the preprint in perpetuity. It is made available under a CC-BY 4.0 International license.

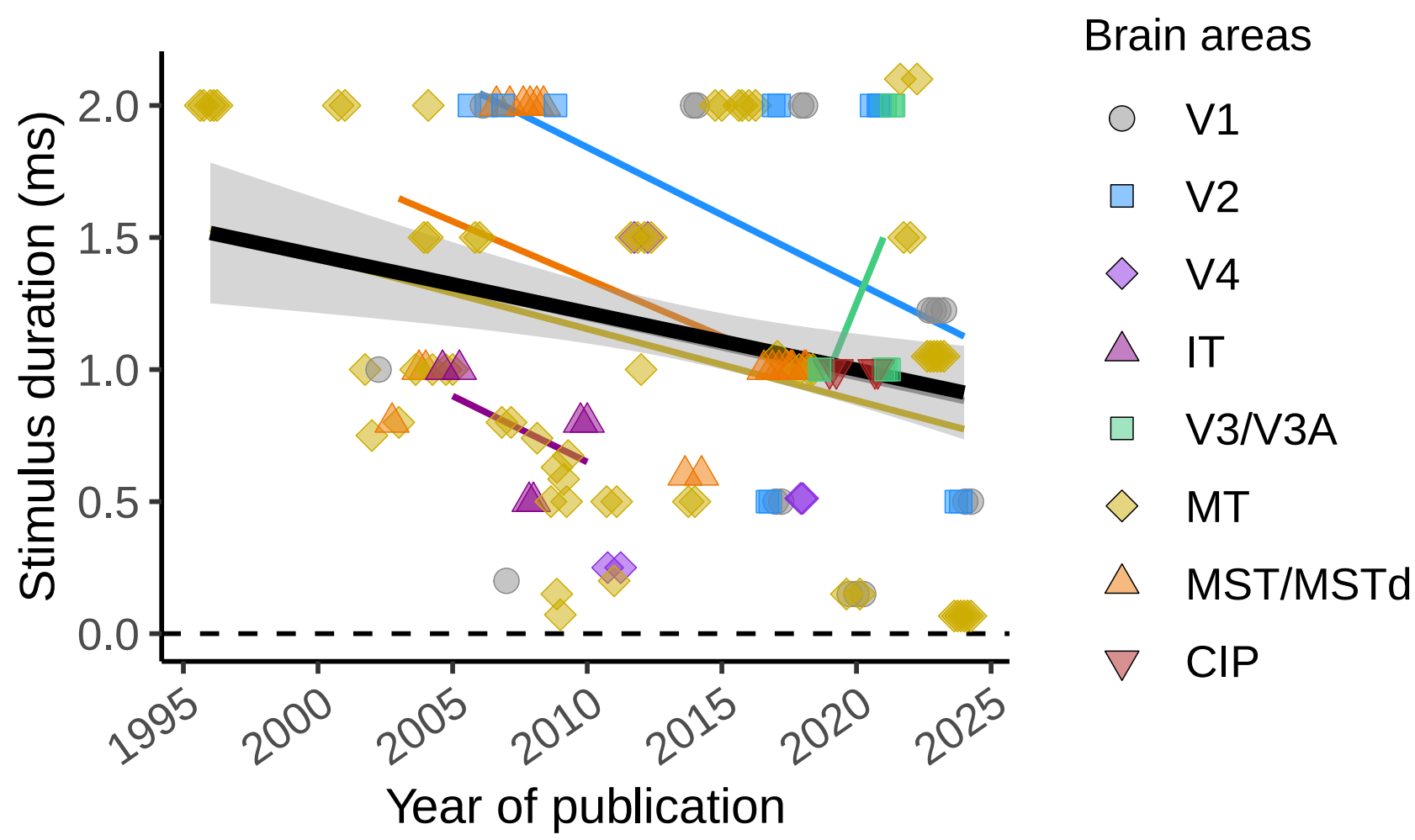

Figure S26: **Relationship between stimulus duration and year of publication.**

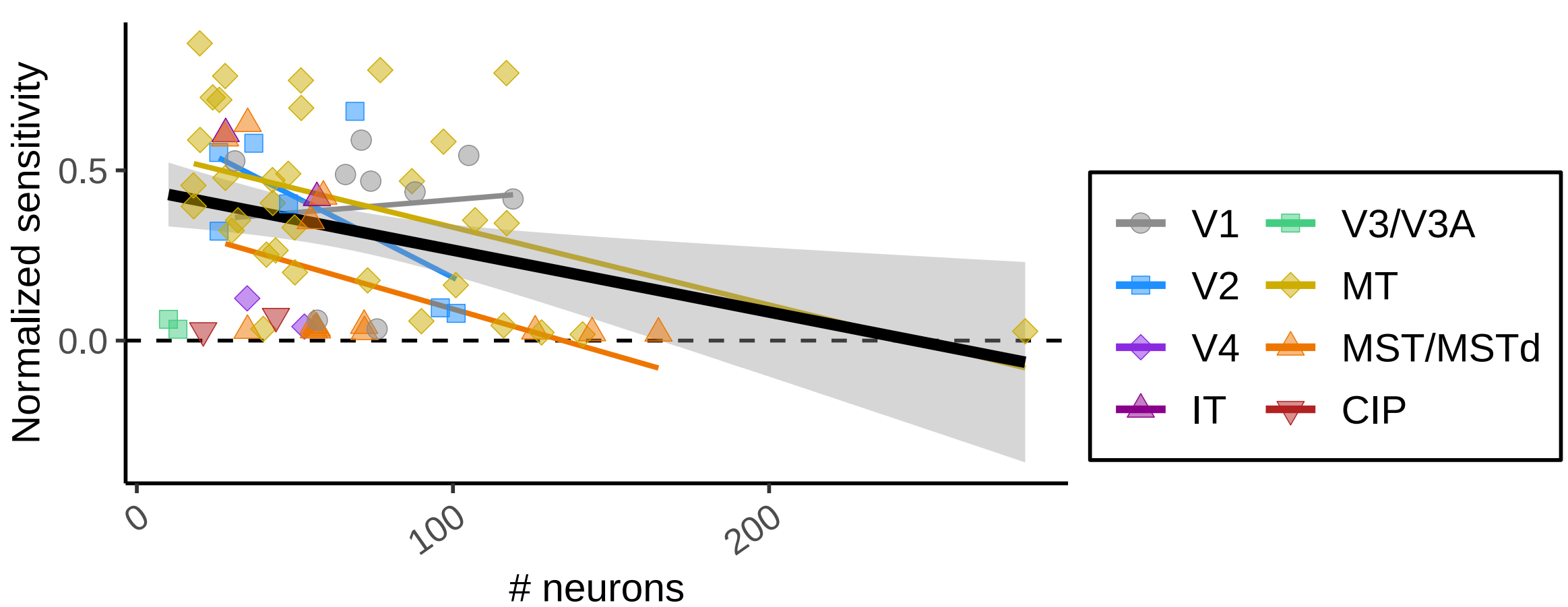

Figure S27: **Relationship between neural sensitivity and number of neurons recorded in the study (only single electrode recordings).**

bioRxiv preprint doi: https://doi.org/10.64898/2026.06.08.730961; this version posted June 11, 2026. The copyright holder for this preprint (which was not certified by peer review) is the author/funder, who has granted bioRxiv a license to display the preprint in perpetuity. It is made available under a CC-BY 4.0 International license.

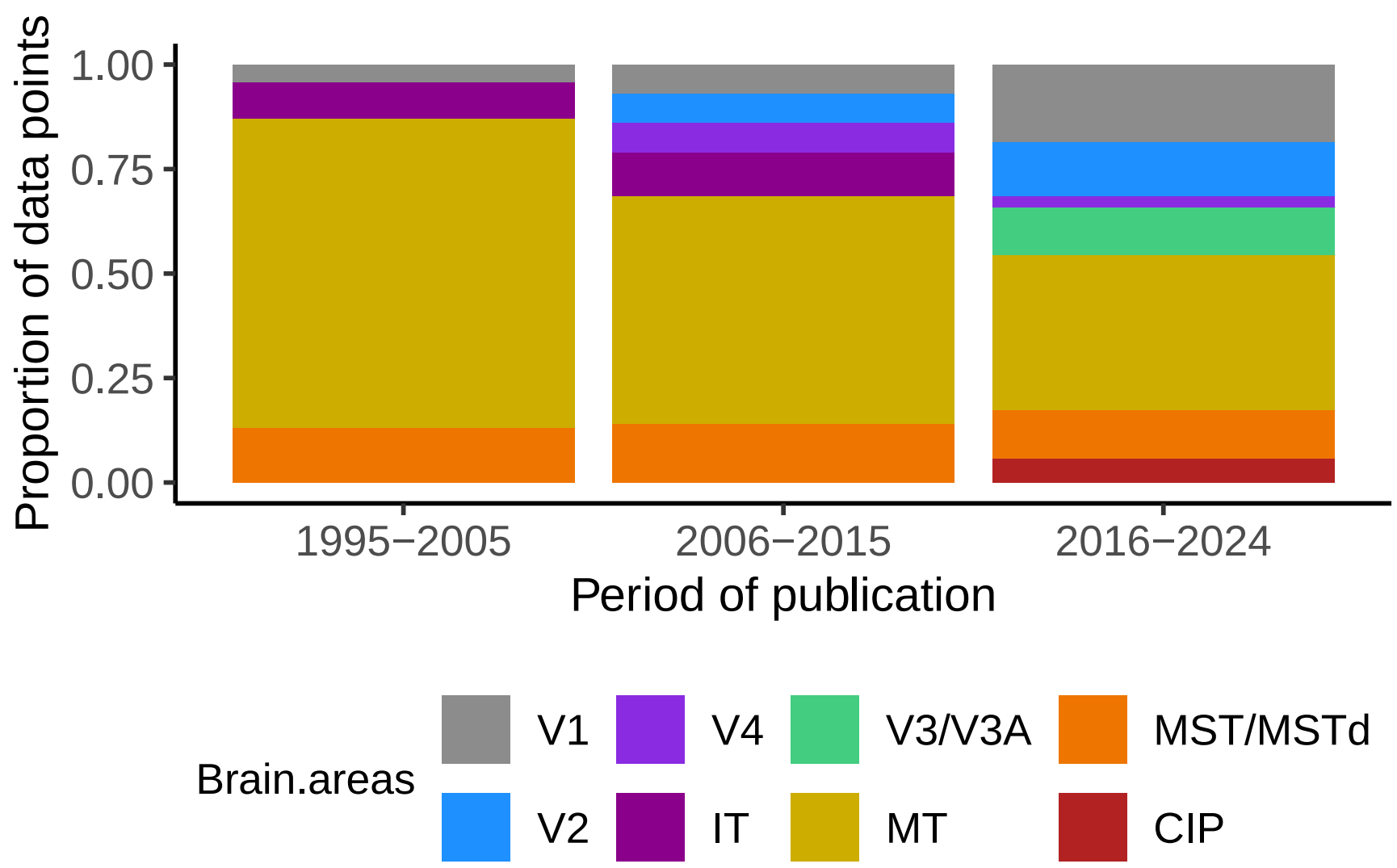

Figure S28: **Changes in the distribution of brain areas studied over time.** Proportion of data points from each brain area is shown across three publication periods, illustrating shifts in focus within the CP literature.

8.3 Variables for which no effect on mean CP was found

Both feedforward and feedback models predict that task exposure (the cumulative duration the animal has performed the task) and performance metrics should positively correlate with mean CP. Longer exposure to the task may increase CP by aligning the animal's strategy with the experimental paradigm and inducing perceptual learning (Law and Gold, 2008; Uka et al., 2012; Sanayei et al., 2018). However, we found no significant effect of task exposure on mean CP – neither in a regression including only task exposure ($p = 0.8$, $N = 36$, Fig. S16) nor in models controlling for sensitivity, brain area, stimulus duration, and task type (inverse mean N/P ratio control: $p = 0.2$, $N = 27$; tailoring control: $p = 0.97$, $N = 33$). Performance metrics that capture how well the monkey engages with the task are also expected to correlate positively with CP. The lapse rate – defined as the error rate on the easiest trials – is widely used as a proxy for such engagement (Prins, 2012; McCarley and Yamani, 2021). A negative correlation with CP was expected (Uka et al., 2012), but no significant relationship was found before (Fig. S17; $p = 0.5$, $N = 37$) and after controlling for four experimental variables (inverse N/P ratio control: $p = 0.5$, $N = 30$; tailoring control: $p = 0.6$, $N = 35$).

Recent work suggests that CP may also be influenced by the spatial arrangement and predictability of saccade targets, through the effects of saccade planning (Laamerad et al., 2024; Zhang and Gu, 2026) (bottom right of Fig. S1). A necessary precondition for the saccade-planning effects is the spatial predictability of saccade targets (Zhang and Gu, 2026). We graded saccade-target predictability across studies on a five-level scale: (1) responses not made by saccades; (2) unpredictable targets; (3) targets varying within a recording day; (4) targets varying between days; and (5) fixed targets across the entire experiment. We found no evidence that target predictability modulated mean CP, but the small number of studies

bioRxiv preprint doi: https://doi.org/10.64898/2026.06.08.730961; this version posted June 11, 2026. The copyright holder for this preprint (which was not certified by peer review) is the author/funder, who has granted bioRxiv a license to display the preprint in perpetuity. It is made available under a CC-BY 4.0 International license.

with low target predictability (no saccades: $N = 8$; unpredictable targets: $N = 6$) limits the strength of this conclusion (Fig. S18).

Researchers have argued that computing CP using trials with non-identical stimuli may introduce confounds that inflate CP estimates (Nienborg and Cumming, 2009; Kang and Maunsell, 2012; Wimmer et al., 2015) (bottom left of Fig. S1). Ideally, CP should be calculated using trials in which the stimulus is fixed and fully ambiguous to avoid contamination from stimulus-driven activity (Britten et al., 1996). In practice, however, most CP studies deviate from this ideal. First, instead of presenting the exact same stimulus across trials (same noise seed, "frozen noise"), researchers often use multiple random noise seeds to prevent monkeys from memorizing specific noise patterns in the stimulus. Following Britten et al. (1996), researchers assumed that the monkey's choice and the neuron's response are not systematically affected by differences in the noise seed. However, Nienborg and Cumming (2009) and Wimmer et al. (2015) showed that this practice can lead to a modest overestimation of CP. Second, most studies compute what is known as "grand CP", in which neuronal responses are z-scored within each stimulus condition and then pooled across all conditions. This approach substantially increases the number of trials available for CP estimation compared to analyses restricted to ambiguous stimuli ("zero signal" in Fig. 2) alone. It rests on the assumption that z-scoring effectively removes all task-relevant stimulus information from the neural responses – otherwise, CP may be artificially inflated due to a stimulus-choice correlation. Conversely, Kang and Maunsell (2012) showed that when choice distributions are highly imbalanced across conditions, z-scoring can underestimate CP by subtracting away part of the choice-related signal. In our meta-analysis, we used grand CP with random seeds whenever possible, as it was the most commonly reported metric across studies (63% of data points, see Section 7). For the subset of studies that reported multiple CP metrics, we directly compared the different methods. We found no significant difference between frozen and random seed conditions (Wilcoxon signed-rank test: $p = 0.2$, $N_{\mathrm{pairs}} = 9$; Fig. S19), nor between grand CP and CP calculated from ambiguous stimulus trials ($p = 0.5$, $N_{\mathrm{pairs}} = 22$; Fig. S20). Overall, our results suggest that while CP calculation methods may introduce some variability, in practice they have little effect on mean CP compared to the much greater uncertainty arising from limited trials and neurons.

If a neuron's stimulus tuning is partially driven by feedback signals, estimating its stimulus preference from task trials can increase CP estimates, particularly for low-sensitivity neurons (Zaidel et al., 2017). In CP calculations, the "positive" choice is defined relative to the neuron's preferred stimulus category (see Fig.2). Zaidel et al. (2017) and Elmore et al. (2019) showed that choice-related signals during task performance can shift estimated preferences toward the direction of choice modulation and flip some CPs from below 0.5 to above 0.5, thereby increasing population mean CP. We found no evidence for such an effect; rather, the 26% of data points from studies that derived preferences from task trials exhibited slightly lower mean CP ($\beta = -0.017$, $p = 0.05$; Fig. S21), a trend that became non-significant when controlling for recording technique ($\beta = -0.012$, $p = 0.16$) or when adjusting for sensitivity, brain area, duration, and task type (inverse mean N/P ratio: $\beta = 0.015$, $p = 0.19$; tailoring: $\beta = -0.004$, $p = 0.7$).

bioRxiv preprint doi: https://doi.org/10.64898/2026.06.08.730961; this version posted June 11, 2026. The copyright holder for this preprint (which was not certified by peer review) is the author/funder, who has granted bioRxiv a license to display the preprint in perpetuity. It is made available under a CC-BY 4.0 International license.

8.4 Variables that do not have good theoretical explanation

The apparent effect of recording technique on mean CP is fully accounted for by differences in sensitivity, brain area, stimulus duration, and task type. Of the 70 data points reporting a mean N/P ratio, only one came from a study using multi-electrode recordings, making it infeasible to assess the recording technique effect using this sensitivity measure. When controlling for tailoring technique alone, the effect of multi-electrode recordings on mean CP was not statistically significant ($\beta_{\text{multi-electrode}} = -0.004$, $p = 0.8$), and it remained non-significant after additionally controlling for brain area, stimulus duration, and task type ($\beta_{\text{multi-electrode}} = 0.007$, $p = 0.6$). Our results suggest that the apparent effect of recording technique on mean CP is driven by tailoring limitations, as multi-electrode studies cannot fine-tune stimuli to individual neurons.

Similarly, the relationships of mean CP with year of publication and number of neurons – patterns suggestive of publication bias – can instead be explained by four key experimental factors. When controlling for neuronal sensitivity, brain area, stimulus duration, and task type, the effect of publication year disappears entirely (inverse mean N/P ratio: $\beta_{\text{year}} = 0.0005$, $p = 0.55$; tailoring-based sensitivity: $\beta_{\text{year}} = -0.0003$, $p = 0.68$). This pattern reflects a temporal decline in both sensitivity and stimulus duration, along with a shift in brain areas studied – from MT to V1 in particular (Fig.3c, S26, S28). The negative association between mean CP and the number of neurons loses statistical significance when accounting for the four variables (single-electrode studies only; inverse mean N/P ratio: $p = 0.15$; tailoring: $p = 0.12$). This attenuation appears primarily driven by a decrease in neuronal sensitivity in studies that recorded from more neurons, as such studies often reduce tailoring efforts due to practical constraints (single-electrode studies only, Fig.S27).

Stimulus eccentricity – the distance between the stimulus and the fixation point – showed no significant relationship with mean CP in any analysis (Fig. S25; pairwise regression: $p = 0.32$, $N = 57$; regression controlling for sensitivity using the inverse N/P ratio: $p = 0.5$, $N = 35$; tailoring-based sensitivity control: $p = 0.3$, $N = 57$).

8.5 Studies with exceptionally high or low mean CP values not explained by experimental variables

We examined data points that appeared as outliers—defined as having the largest residuals—in our main regression models that included neuronal sensitivity (either inverse mean N/P ratio or tailoring-based proxy), brain area, stimulus duration, and task type.

Several studies demonstrated pronounced heterogeneity between monkeys, with mean CP values for one animal showing extreme deviations from model predictions. Britten et al. (1996) reported a relatively low mean CP in MT for one of four monkeys (monkey J: mean CP = 0.51, prediction error in model with inverse mean N/P ratio = –2.2 SD), despite a large sample size ($N = 77$; CP values for the other monkeys: 0.53, 0.57, 0.58). An even more striking outlier comes from Goris et al. (2017), who reported a mean CP below 0.5 and a negative CP–sensitivity relationship in V2 for one monkey (monkey 1: mean CP = 0.46, $N = 48$, prediction error in model with inverse mean N/P ratio = –1.8 SD; model with tailoring = –1.9 SD), in contrast to the other monkey in the study (mean CP = 0.53). Uka and DeAngelis (2006) observed a similar pattern in area MT: one monkey

bioRxiv preprint doi: https://doi.org/10.64898/2026.06.08.730961; this version posted June 11, 2026. The copyright holder for this preprint (which was not certified by peer review) is the author/funder, who has granted bioRxiv a license to display the preprint in perpetuity. It is made available under a CC-BY 4.0 International license.

(monkey B) had a noticeably low mean CP of 0.47 ($N = 48$, prediction error in model with inverse mean N/P ratio = –2.6 SD; in model with tailoring = –2.1 SD), whereas the other monkey exhibited a mean CP of 0.57, a value typical for this brain area. Such pronounced inter-animal variability suggests that individual task strategies or the sampled neuronal populations are more heterogeneous than is assumed by theoretical models.

Some studies using bistable stimuli reported either unexpectedly high or low mean CP values. Maier et al. (2007) reported high CPs in MT with binocular motion rivalry stimuli, despite using much shorter duration of 800 ms compared to the 2000 ms typical for rotating cylinder tasks and tailoring stimulus size for a multi-neuron population rather than individual units (two monkeys combined: mean CP = 0.68, prediction error in model with tailoring = +2.1 SD). In this study, monkeys did not directly report their percepts; instead, perception was inferred via binocular flash suppression—a technique where presenting a pattern to one eye and subsequently flashing a dissimilar pattern to the other reliably biases perception toward the newly flashed stimulus. By comparison, Williams et al. (2003) found no significant CP in MT using a bistable apparent motion stimulus where evenly spaced columns of dots abruptly jump by half their inter-column distance, creating an illusion of movement in one of two opposite directions (two monkeys combined: mean CP = 0.50, prediction error in model with tailoring as sensitivity control = –2.8 SD). The authors argued that, unlike the rotating cylinder, their stimulus was suboptimal for MT and may have engaged higher-order areas. Similarly, Grunewald et al. (2002) found that the rotating cylinder stimulus failed to elicit significant CP in V1, suggesting that, unlike MT, this area does not contribute to the perception of such complex stimuli (two monkeys combined: mean CP = 0.48, prediction error in model with tailoring as sensitivity control = –2.2 SD).

Ghose and Harrison (2009) used a very brief stimulus presentation (60–83 ms) in a detection task but nevertheless reported high CP values in MT (two monkeys combined: mean CP = 0.64, prediction error in model with tailoring = +2.5 SD). The study employed a non-standard stimulus for MT: an array of 31 small Gabor patches that began drifting coherently at a specific point in time. A second array was presented symmetrically outside the neurons' receptive fields, so the monkey could not predict which array would be behaviorally relevant on a given trial. Another unusual feature was that the stimuli were retinally stabilized to correct for fixational eye movements. Additionally, CP was estimated with a non-standard method that adjusted latency and time windows individually for each neuron to maximize CP values. Interestingly, and in contrast to our meta-analysis findings, the authors attributed the elevated CPs to the short stimulus duration – arguing that the highly informative, spatially compact, and temporally precise stimulus elicited strong, low-latency responses from MT neurons.

Palmer et al. (2007) reported an unusually high mean CP in V1 during a detection task (three monkeys combined: mean CP = 0.62; prediction error in model with inverse mean N/P ratio = +2.5 SD, model with tailoring = +2.8 SD). Despite these elevated CP values, no significant correlation between CP and neuronal sensitivity was found. In this study, monkeys were required to detect a low-contrast Gabor patch. Interestingly, the elevated mean CP values were observed only for trials in which the stimulus was present (hits vs. misses), which we chose to use in our meta-analysis. In contrast, trials without a stimulus (false alarms vs. correct rejections) showed much lower CP (mean = 0.52, we did not used this value in our analysis). The authors partly attributed this discrepancy to premature

bioRxiv preprint doi: https://doi.org/10.64898/2026.06.08.730961; this version posted June 11, 2026. The copyright holder for this preprint (which was not certified by peer review) is the author/funder, who has granted bioRxiv a license to display the preprint in perpetuity. It is made available under a CC-BY 4.0 International license.

saccades and "fast guesses" during false alarm trials and also suggested that in the absence of a stimulus and low activity in V1, decision variability arises primarily from downstream noise sources.

8.6 On the Difference Between Mean P/N Ratio and Inverse Mean N/P Ratio

To clarify the difference between the mean P/N ratio and the inverse mean N/P ratio used in our analyses, we consider a simplified notation. Let the psychometric threshold P, which tends to be relatively constant across neurons within an experiment (especially after training), be replaced with a constant a. In contrast, let the neurometric threshold N, which varies across neurons, be denoted by the random variable x. This substitution allows us to isolate the effect of nonlinearity when aggregating across neurons.

Nonlinearity of Expectation and Reciprocals

Consider the following two expressions:

$$\mathbb{E}\left[\frac{a}{x}\right] \quad \text{and} \quad \frac{1}{\mathbb{E}\left[\frac{x}{a}\right]}.$$

These two quantities are generally not equal and are not linearly related. This is a direct consequence of the convexity of the reciprocal function and the nonlinearity of expectation.

Specifically, Jensen's inequality implies:

$$\mathbb{E}\left[\frac{a}{x}\right] \geq \frac{a}{\mathbb{E}[x]},$$

and similarly,

$$\mathbb{E}\left[\frac{x}{a}\right] \geq \frac{1}{\mathbb{E}\left[\frac{a}{x}\right]}.$$

Therefore, it follows that:

$$\mathbb{E}\left[\frac{a}{x}\right] > \frac{1}{\mathbb{E}\left[\frac{x}{a}\right]},$$

unless x is constant. Intuitively, when x takes small values (i.e., when neurons have high sensitivity), the reciprocal $\frac{a}{x}$ becomes large, which skews the expectation upward. This distinction is critical, as aggregating P/N vs. N/P ratios across neurons will yield systematically different results.

Implications for Regression Analysis

Let us now consider two linear regressions without intercept relating a dependent variable y to the two different versions of the sensitivity ratio:

$$(1)\ y \sim \mathbb{E}\left[\frac{a}{x}\right] \quad \text{with slope } \beta,$$
$$(2)\ y \sim \frac{1}{\mathbb{E}\left[\frac{x}{a}\right]} \quad \text{with slope } \beta'.$$

bioRxiv preprint doi: https://doi.org/10.64898/2026.06.08.730961; this version posted June 11, 2026. The copyright holder for this preprint (which was not certified by peer review) is the author/funder, who has granted bioRxiv a license to display the preprint in perpetuity. It is made available under a CC-BY 4.0 International license.

Because $\frac{1}{\mathbb{E}\left[\frac{x}{a}\right]} < \mathbb{E}\left[\frac{a}{x}\right]$, the independent variable in regression (2) tends to be smaller in magnitude. To achieve a similar fit to the dependent variable y, the model compensates with a larger slope. Thus, we typically find:

$$\beta' > \beta.$$

This means that using the inverse mean N/P ratio as a predictor (as done in some of our control analyses) tends to produce larger regression slopes compared to using the mean P/N ratio.
